# Evaluation of a fast-and-frugal clinical decision algorithm (‘pathways’) on clinical outcomes in hospitalised patients with COVID-19 treated with anticoagulants

**DOI:** 10.1111/jep.13780

**Published:** 2022-10-13

**Authors:** Benjamin Djulbegovic, Iztok Hozo, David Lizarraga, Joseph Thomas, Michael Barbee, Nupur Shah, Tyler Rubeor, Jordan Dale, Jochen Reiser, Gordon Guyatt

**Affiliations:** 1 Department of Computational & Quantitative Medicine, City of Hope, Beckman Research Institute, Duarte, California, USA; 2 Division of Health Analytics, Beckman Research Institute, Duarte, California, USA; 3 Evidence-Based Medicine & Comparative Effectiveness Research, Beckman Research Institute, Duarte, California, USA; 4 Department of Mathematics, Indiana University, Gary, Indiana, USA; 5 Rush University Medical Center (RUMC), Chicago, Illinois, USA; 6 Division of Hospital Medicine, Department of Hospital Medicine, Rush University Medical Center, Chicago, Illinois, USA; 7 Department of Emergency Medicine, Rush University Medical Center, Chicago, Illinois, USA; 8 Houston Methodist Academic Institute, Houston, Texas, USA; 9 Department of Internal Medicine, Rush University Medical Center, Chicago, Illinois, USA; 10 Department of Health Research Methods, Evidence, and Impact, McMaster University, Hamilton, ON, Canada

**Keywords:** clinical decision making, clinical pathways, decision support, evidence based medicine, fast-and-frugal trees, practice guidelines

## Abstract

**Rationale, Aims and Objectives:**

Critics have charged that evidence-based medicine (EBM) overemphasises algorithmic rules over unstructured clinical experience and intuition, but the role of structured decision support systems in improving health outcomes remains uncertain. We aim to assess if delivery of anticoagulant prophylaxis in hospitalised patients with COVID-19 according to an algorithm based on evidence-based clinical practice guideline (CPG) improved clinical outcomes compared with administration of anticoagulant treatment given at individual practitioners’ discretion.

**Methods:**

An observational design consisting of the analysis of all acutely ill, consecutive patients (*n* = 1783) with confirmed COVID-19 diagnosis admitted between 10 March 2020 to 11 January 2022 to an US academic center. American Society of Haematology CPG for anticoagulant prophylaxis in hospitalised patients with COVID-19 was converted into a clinical pathway and translated into fast-and-frugal decision (FFT) tree (‘algorithm’). We compared delivery of anticoagulant prophylaxis in hospitalised patients with COVID-19 according to the FFT algorithm with administration of anticoagulant treatment given at individual practitioners’ discretion.

**Results:**

In an adjusted analysis, using combination of Lasso (least absolute shrinkage and selection operator) and propensity score based weighting [augmented inverse-probability weighting] statistical techniques controlling for cluster data, the algorithm did not reduce death, venous thromboembolism, or major bleeding, but helped avoid longer hospital stay [number of patients needed to be treated (NNT) = 40 (95% CI: 23–143), indicating that for every 40 patients (23–143) managed on FFT algorithm, one avoided staying in hospital longer than 10 days] and averted admission to intensive-care unit (ICU) [NNT = 19 (95% CI: 13–40)]. All model’s selected covariates were well balanced. The results remained robust to sensitivity analyses used to test the stability of the findings.

**Conclusions:**

When delivered using a structured FFT algorithm, CPG shortened the hospital stay and help avoided admission to ICU, but it did not affect other relevant outcomes.

## 1 | INTRODUCTION

It is generally considered that interventions delivered according to evidence-based clinical practice guidelines (CPGs) improve health outcomes.^1,2^ However, empirical evidence supporting this widely held belief is limited, prompting the critique that evidence-based medicine (EBM) overemphasises algorithmic decision rules over physicians’ intuition and experience.^3,4^

During the COVID-19 pandemic many inadequately tested and unproven therapies have, largely driven by uncontrolled physicians’ experience, dominated the practice of medicine^5,6^ raising the question if health interventions delivered according to evidence-protocols could have improved health outcomes.^7^

One of the challenges of evaluating evidence-based practices is the lack of a theoretical framework for their evaluation.^8,9^ Clinical practice mostly entails a series of decisions, while CPGs usually consists of single or multiple recommendations that typically are not linked via a series of decisions into a coherent management strategy. Clinical pathways (CPs) can help logically organise the sequence of clinically decisions.^10,11^ That is, CPGs can be thought of as addressing one recommendation at a time,^12^ while CPs represent healthcare plans (referred to as protocols, clinical algorithms, or flow-charts) that provide detailed steps about the course of management of a particular clinical problem or the entire spectrum of care.^11,12^

Although it is estimated that CPs are implemented in more than 80% of hospitals in the United States,^10,11,13^ they are also theory-free constructs, typically developed in an ad hoc manner adhering in varying degrees to evidence-based practices.^9^ CPs can, however, be translated into fast-and-frugal (FFT) decisions trees- sound theoretical constructs that allow the quantitative analysis of delivery healthcare interventions.^9,14^ FFT draws its theoretical robustness by relating to signal detection theory, evidence accumulation theory, and the threshold model to help improve decision-making.^14,15^ FFTs consist of simple decision trees composed of sequentially ordered cues (tests) and binary (yes/no) decisions formulated via a series of *If–then* statements.^14^ Decision strategies based on FFTs have been found to be superior to other decision and classification strategies, including those using complex multivariate regression and machine learning models.^15,16^ FFTs are grounded in the *heuristic approach* to rational decision making.^16–20^ Importantly, both clinical practice and medical education relies on heuristics as one of the key problem solving and decision making strategies.

COVID-19 is a thromboinflammatory disorder, which places infected patients with SARS-Cov-2 virus at risk for venous-thromboembolism (VTE), bleeding and death.^21,22^ The risk is further amplified in hospitalised patients.^21,22 23^ Using GRADE (Grading of Recommendations Assessment, Development and Evaluation)—the state-of-the-art system for developing CPGs- the American Society of Haematology (ASH) developed recommendations for anticoagulation of acutely ill patients with COVID-19. In February of 2021, the ASH panel issued weak/conditional recommendations (suggestions) in favour of prophylactic anticoagulation,^24^ while in May of 2022,^25^ the panel updated their recommendations to suggest the use of therapeutic intensity anticoagulation for acutely but not critically ill hospitalised patients with COVID-19.

Prevention of the aforementioned COVID-19 complications can be instituted according to the FFT-based decision tree, or be left to individual providers’ discretion (‘usual care’). Therefore, we set out to evaluate if adherence to evidence-informed clinical pathway translated into FFT compared with ad hoc administration of anticoagulant as per individual clinicians’ choice results in improved patients’ outcomes.

## 2 | METHODS

### 2.1 | Eligible patients

All acutely ill, consecutive patients with confirmed COVID-19 diagnosis not requiring admission to an intensive care unit (ICU) were eligible for the analyses. The patients were admitted from 10 March 2020 to 11 January 2022 to one of the three Rush hospitals in Chicago.

### 2.2 | Evidence and guidelines

Based on comprehensive review of mostly non-randomised evidence, the ASH panel initially issued weak/conditional recommendations (suggestions) in favour of prophylactic anticoagulation using low-molecular weight heparins (LMWH).^24^ After consideration of evidence from additional six randomised trials (RCTs)—two on the effects of direct acting anticoagulant (DOAC) rivaroxaban and four related to prophylaxis with LMWH,^26–31^ the panel updated its recommendation in May of 2022^25^ in favour of therapeutic intensity anticoagulation with LMWH for acutely but not critically ill hospitalised patients with COVID-19. The recommendations are consistent with two meta-analyses of RCTs, which indicated beneficial effects of therapeutic intensity anticoagulation in terms of reduction of VTE but no significant effect on overall mortality and major bleeding.^32,33^ However, on both occasions, the panel assessed that recommendations was based on very low certainty in the evidence, acknowledging that administration of the prophylactic LMWH is also appropriate, particularly in patients considered at the lower risk^34^ for COVID-19 complications or higher bleeding risk.^24,25^ The latter is also echoed by International Society on Thrombosis and Haemostasis (ISTH), which issued a strong recommendation for the use of prophylactic over therapeutic anticoagulation for non–critically ill hospitalised patients with COVID-19.^35^

### 2.3 | Pathway-FFT algorithm

**FIGURE 1:**
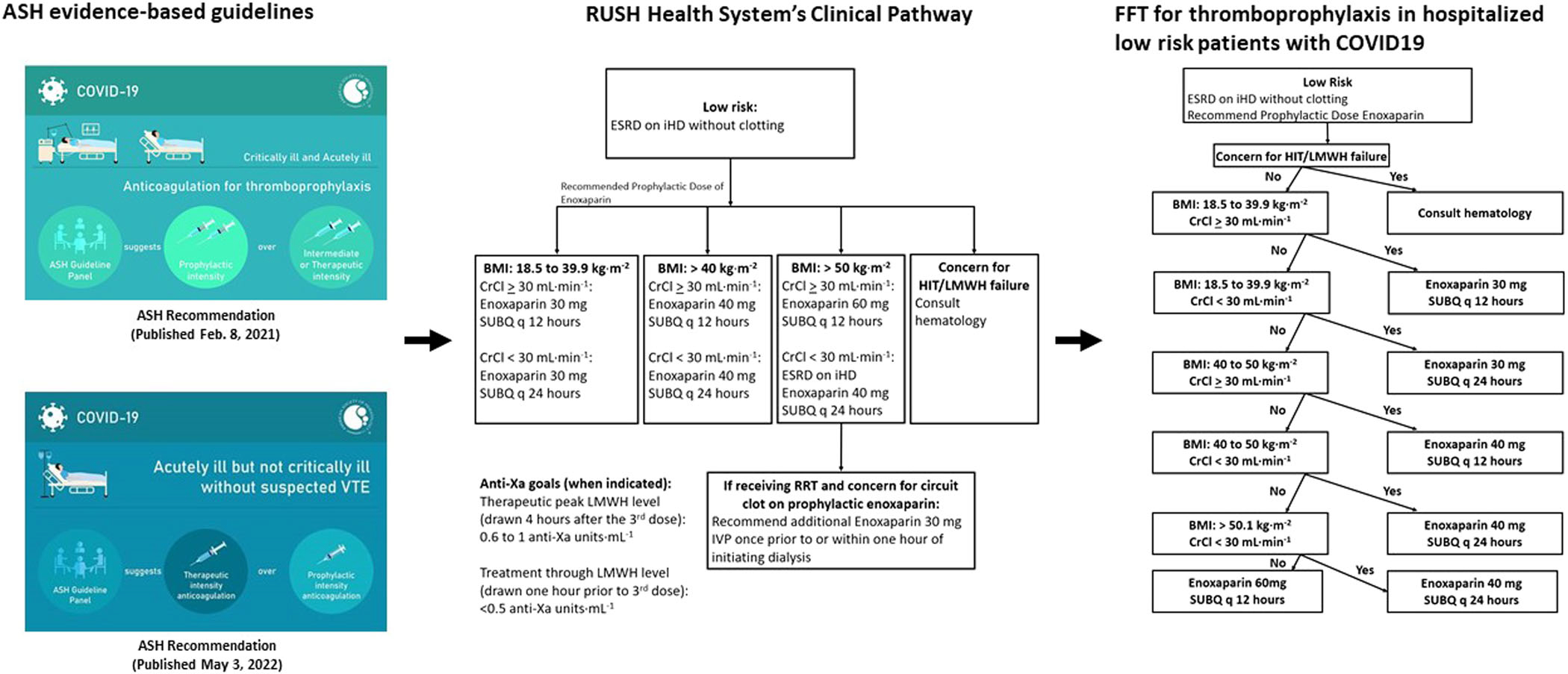
A visual presentation of a conversion of the American Society of Haematology evidence-based guidelines into Rush hospital clinical pathways and their translation into fast-and-frugal (FFT) decision tree

Figure 1 visually presents conversion of ASH CPGs into CPs, and their translation into FFT. One of the challenges of developing evidence-based management for COVID-19 is the rapid change in evidence base requiring continuous update of the guidelines.^36^ Given the quick realisation that COVID-19 is a thromboinflammatory disorder, the Rush Health System has developed pathways endorsing prophylactic anticoagulation for low risk hospitalised patients, initially based on observational data. Periodic re-assessment of evidence base related to antithrombotic treatment in COVID-19, including the ASH and other evidence-based guidelines indicated no need to change the pathways. Therefore, Figure 1 represents FFT based on best existing evidence as of January 2022 (when the data collection was locked).

Given uncertainty about evidence and weak recommendations, Rush has also allowed treatment off pathways per discretion of individual clinicians. FFT-based strategy has solely employed prophylactic anticoagulation with LMWH but according to standard, evidence-based dose adjustments and the protocols.^37,38^ A management off pathway has typically been ad hoc, not strictly protocolized even though it may have included the same type of drugs. Note that the management according to the pathway was delivered via standard orders, which we then (retrospectively) converted into FFTs to allow the proposed analysis. Importantly, it is FFT’s clear and explicit ‘*If–then*’ rules that allow accurate assessment of adherence to the FFT/pathway intervention to distinguish between those patients who received prophylactic anticoagulation in the control (‘off-pathway’) versus on pathway group. Fundamentally, because the pathway is composed of built-in standard orders, the adherence to the FFT-based management guarantee better compliance with the treatment, which makes us hypothesise that the improved outcomes will be observed in on vs off-pathway arm. *Because our main goal is to contrast two management strategies, we aimed to compare the use of evidence-based pathway (algorithm) versus not adhering to FFT-pathway (Figure 1), while having patients in both groups received prophylactic anticoagulation.* Thus, two groups were identical according to all covariates except the management received.

### 2.4 | Outcomes

Because the use of composite endpoints depends on a common biology, similar relative effects, and similar importance—and without these commonalities can be misleading.^39^ we selected death, VTE and major bleeding as three key primary outcomes, and hospital length of stay greater than 10 days [LOS10d] and admission to ICU as two secondary outcomes.

### 2.5 | Data collection and validation

We collected data on key demographics, clinical items, treatments, and health outcomes from the Rush electronic medical records (EPIC)/discharge records. Supporting Information: Appendix 3 shows ICD10 and other codes we used to select the variables of interest for the analysis. We considered that outcomes death, LOS and admission to ICU were accurately recorded in the electronic record/administrative data sets we used. We further reviewed the pharmacy records, orders, progress and discharge notes to ascertain the accuracy of classification for 100% of patients who developed outcome of interest (VTE, major-bleed), and 20% of random cases of the patients deemed not to have VTE or major bleeding. The overall accuracy for classification of VTE outcome was 97% and 94% for major bleed, respectively. Using the same data source, we also assessed the overall accuracy of exposure for delivery of anticoagulants. We determined the overall accuracy of 82% in the ascertainment of the exposure. Eighteen percent of patients who were not correctly classified had their treatment switched from no treatment to therapeutic to prophylactic or vice verse. We investigated the effect of the anticoagulant switch in a sensitivity analysis.

### 2.6 | Statistical analysis

#### 2.6.1 | Rationale

Statistically, one can argue that because its strong theoretical underpinning, evaluation of FFT-algorithm can be assessed using unadjusted comparisons. However, in observational studies we can never be sure that the effects of intervention (adherence to FFT-pathway algorithm, in our case) are solely responsible for observed outcome and not prognostic imbalance or cointerventions. Hence, these factors ought to be considered in the analysis. We therefore performed adjusted analysis by considering covariates that are commonly reported in the literature to affect the outcomes of patients with COVID-19.^40^

Because we could not strongly postulate which of these covariates—with multiple interactions—should be selected in the final adjusted analysis, we used *lasso* (least absolute shrinkage and selection operator) statistical technique to select relevant variables for the analysis while remaining robust to the problem of overfitting; indeed, high-dimensional *lasso* can handle situations when there are more variables than observations. We employed adaptive *lasso* method that not only avoids overselecting the covariates with zero coefficients, but also avoids missing covariates with large coefficients as some other commonly used lasso methods such as cross-validation (CV) or plug-in techniques are prone to do.

Once *lasso* selected the relevant variables, we applied propensity score methods to create a balanced covariate distribution between treated (on FFT-pathway) and untreated (off FFT-algorithm) groups.^41^ We used Stata programme *telasso logit*, which combines *lasso* with propensity score based weighting [augmented inverse-probability weighting (AIPW)] technique, in which we controlled for observations within each Rush Campus Site.^42^ By achieving balance in the covariates between two comparison groups, the AIPW allows estimation of the treatment that is, the decision algorithm’s effect independent of the effects of observed covariates. We expressed the effects of FFT-algorithm in terms of the average treatment effect (ATE), defined as the mean of the difference between two treatment groups (treatment according to FFT clinical algorithm vs. treatment off algorithm). Where appropriate we also expressed the results as NNT (number of patients who would need to be managed by one strategy compared to another to prevent one COVID-19-related outcome).^43^

We tested validity of the model by using a series of diagnostic tests. These consisted of testing the nonviolation of the overlap assumption (requiring an overlap between the treatment and control to meet requirement of exchangeability with respect to all covariates included in the model) and testing for balance in the covariate distribution between treated (on FFT-algorithm) and untreated (off FFT-algorithm) patients. We used Stata *teffects overlap* routine to test for violation of overlap assumption, *tebalance overid* as a global test to reject the null hypothesis that covariates are balanced, *tebalance summarised* to test for balance for each individual covariate and summarise differences between each covariates. We accepted standardised mean differences (SMD) < 0.1 as evidence of well-balanced covariates.^44^

As is typical for any observational study, data on the number of covariates were missing. Therefore, before the analyses, we imputed missing data. After determining that data were missing at random (MAR), we used multiple imputation method that, unlike complete case analysis, does not generate biased results.^45,46^ We used Stata *mi impute chained* routine, which implement multivariate imputation using chained equations (MICE) method.^42^ We conducted five imputations, each of which generated similar results, thus making more imputations unnecessary. We used Rubin’s rules^47^ to combine the imputed data sets into a pooled estimate in the final analysis along with the estimates for each imputed set.

#### 2.6.2 | Sensitivity analyses to assess the robustness of the results

Our default analysis was per intention-to-treat (ITT). Because, as mentioned, some patients had their anticoagulants switched (AC switch) from none to prophylactic to therapeutic and vice versa, to assess the impact of this switch in the exposure, we also performed sensitivity analysis by dropping these patients from the analysis. For four variables (CK, LDH, BNP and d-dimer), imputed data exceeded 50%; hence, we performed sensitivity analyses by repeating all analyses with imputed more than 30% of missing data. We report the analysis according to STROBE (Strengthening the Reporting of Observational studies in Epidemiology) guidelines.^48^

Because this project is considered a quality improvement project, it was deemed by the COH Institutional Board Review (IRB) to require no formal ethical review/approval [COH Protocol #/Ref #:/,20646,200278]. However, high research standard to protect confidentiality and guard against breach of privacy has been exercised, and no patient identifiers have been used/made available to the authors.

## 3 | RESULTS

**FIGURE 2:**
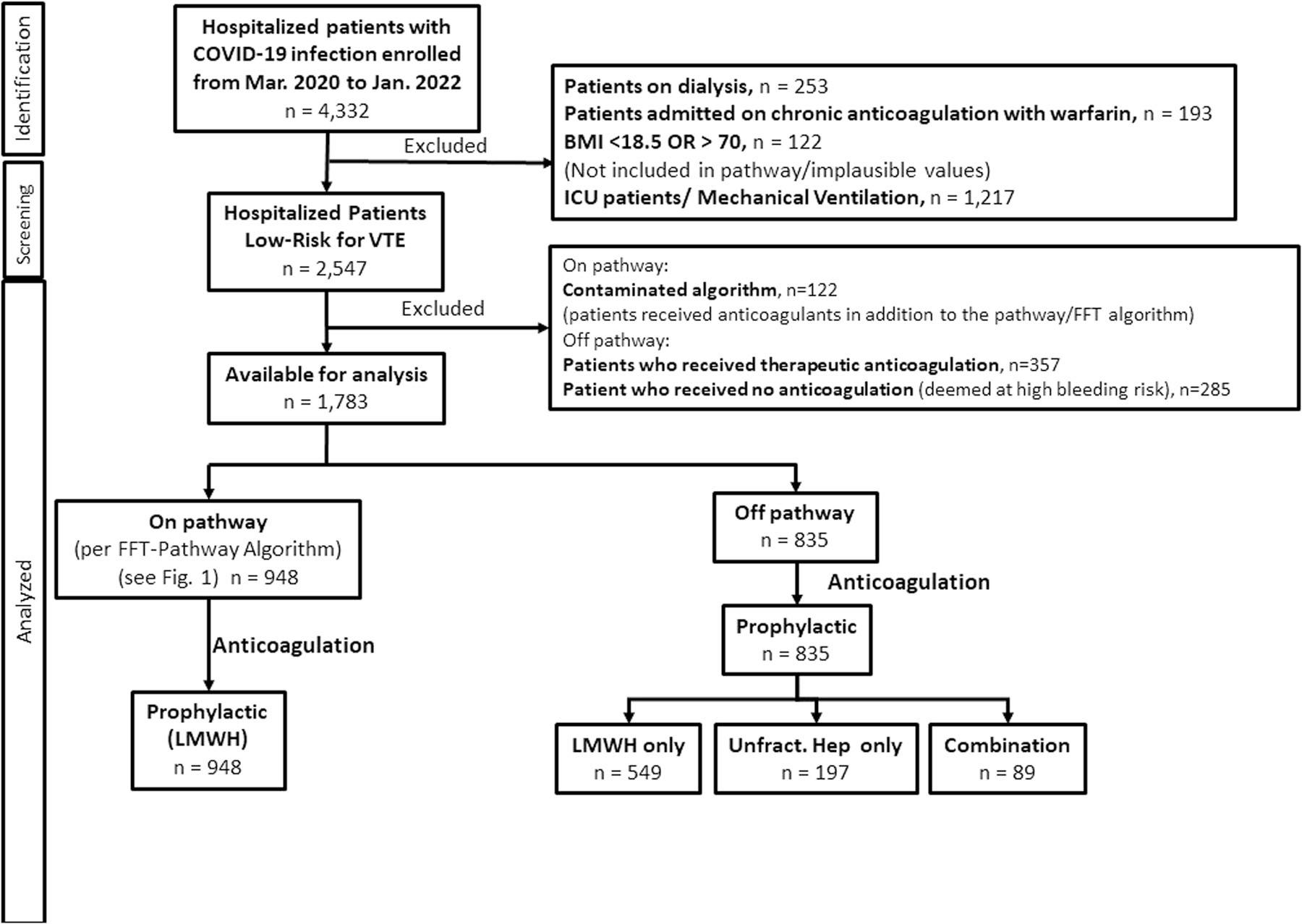
STROBE flow-chart depicting the study patients’ enrolment, exclusion criteria and data availability for the analysis according to anticoagulation treatment. Abbrevations: unfract.: unfractionated.

Figure 2 shows the STROBE flow-chart depicting the study patients’ enrolment, exclusion criteria and data availability for the analysis according to anticoagulation treatment. From 10 March 2020 to 11 January 2022, 4332 consecutive patients diagnosed with COVID-19 were admitted to three hospitals within the Rush Health System. Of these, 2549 were excluded, leaving 1783 patients for the analyses. All remaining patients received prophylactic anticoagulation, 948 (53%) according to pathway/FFT algorithm, and 835 (47%) off pathway, per individual providers’ discretion. All patients managed on pathway received LMWH enoxaparin. Of 835 patients treated off pathway, 549 (66%) were given LMWH (enoxaparin), 197 (24%) received unfractionated heparins and 89 (10%) got ‘combined’ treatment (typically DOAC (direct oral anticoagulants) followed by unfractionated heparins).

Table 1 shows variables considered in the analysis. Sixty-one percent of patients had mild or no comorbidities; 24% moderate and 15% of patients had severe Charlson Comorbidity Index ≥5.^49^ More than 70% of the patients required oxygen supplementation on admission. Between 1/3 to 2/3 patients received antibiotics, antiviral agents (including remdesivir) and steroids.

Most variables were not balanced making the analysis based on unadjusted comparison potentially biased.

**FIGURE 3:**
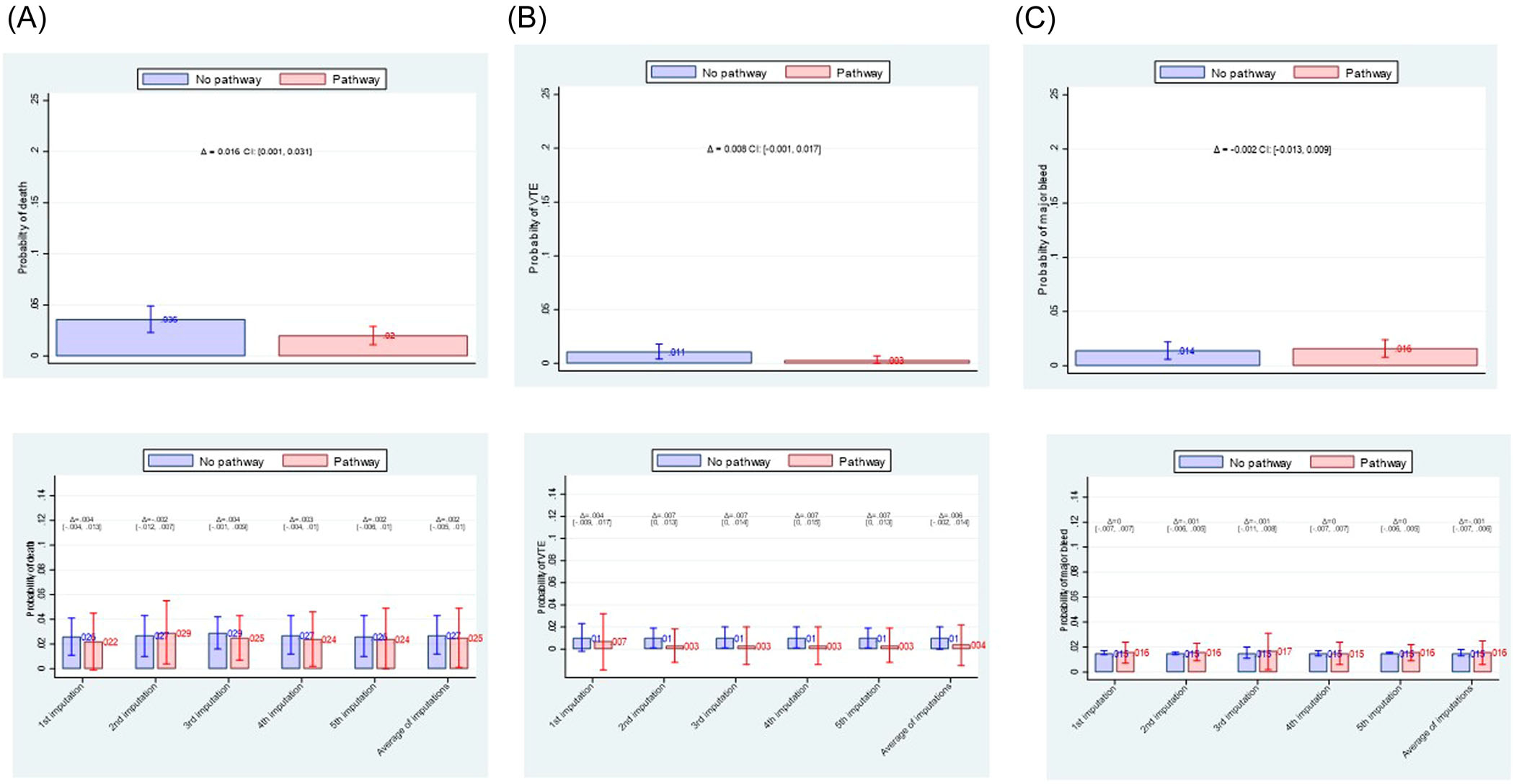
Comparison of effect of fast-and-frugal decision (FFT)-algorithm versus management off the algorithm on reducing death, VTE (venous thromboembolism) or major bleeding. (A) unadjusted analysis (upper row); (B) adjusted analysis (lower row). Overall, there is no difference in effects between two management strategies on any of clinical outcomes.

**FIGURE 4:**
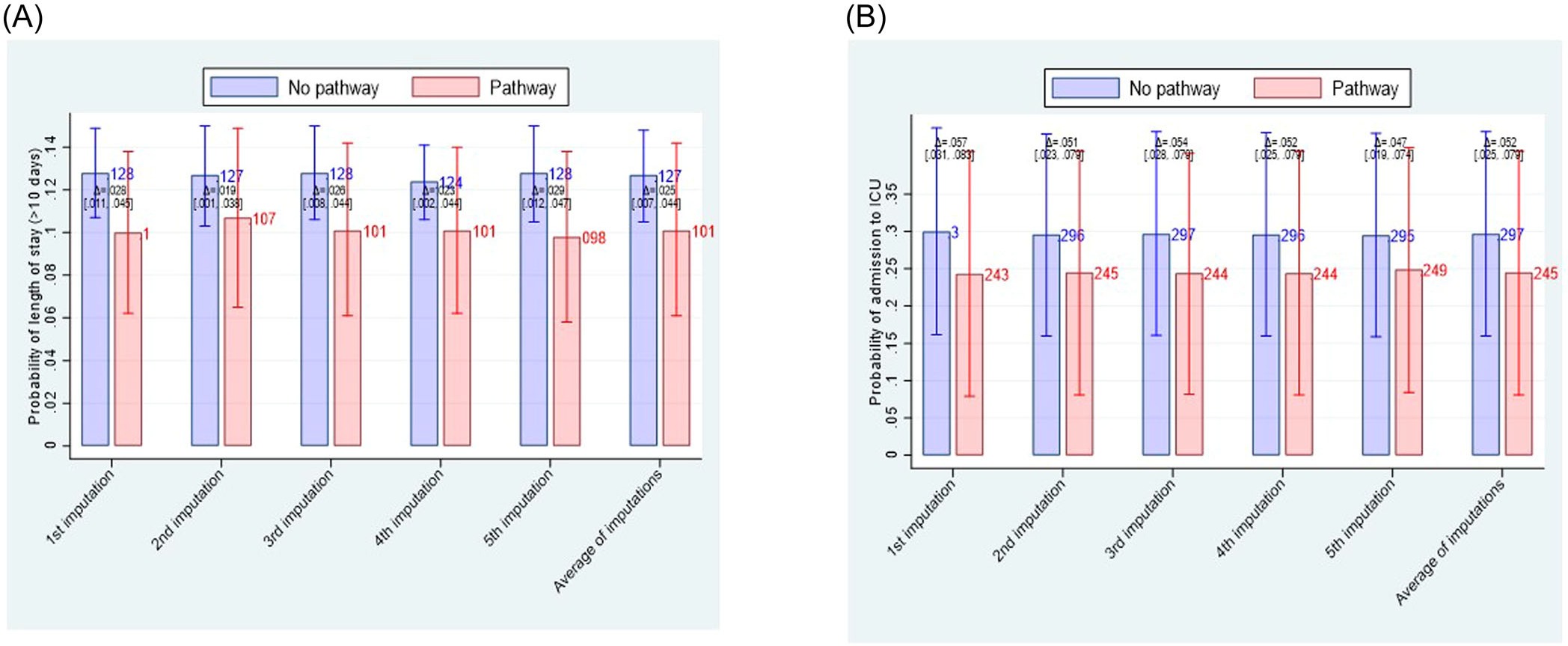
Comparison of effect of fast-and-frugal decision (FFT)-algorithm versus management off the algorithm on secondary outcomes. Compared with the usual care, FFT-based strategy helped avoided stay in the hospital longer than 10 days by about 2.5% (95% CI: 0.7%–4%) (A) and averted admission to intensive-care unit (ICU) by about 5% (95% CI 2.5%–8%) (B).

Figure 3 shows the results of an unadjusted and adjusted analysis. While unadjusted analysis suggested that the management according to the algorithm may reduce mortality compared to treatment off algorithm, this was not confirmed in the adjusted analysis [ARR = 0.2% (95% CI: −0.5% to 1%]. Adjusted analysis show no difference in VTE [ARR = 0.6% (95% CI: −0.2% to 1.4%] and major bleeding rate [ARR = 0.1% (95% CI: −0.7% to 0.6%]. The management according to pathway/FFT algorithm helped avoid a longer hospital stay: NNT = 40 (95% CI: 23–143), indicating that for every 40 patients (23–143) managed on FFT algorithm, one avoided staying in hospital longer than 10 days, while one in 19 (95% CI: 13–40) averted admission to ICU (Figure 4).

Supporting Information: Tables A1–A3 show output of *telasso* regression analysis for all primary outcomes. Out of 28 postulated variables (Table 1) that may be associated with outcomes of interest, *lasso* created 127 potentially relevant variables (including interactions) to ultimately select between 9 and 32 variables, depending on outcome (Supporting Information: Tables A1–A3).

Supporting Information: Figures A1 to A5 show standardised differences before and after propensity score weighting. All variables retained in the analyses were well balanced with SMD < 0.1. Similarly, we detected no violation of overlap assumption.

Sensitivity analysis based on dropping variables with >30% of missing data Supporting Information: Figures 6A to 8B) and per actual treatment received that is, when patients with AC switch were dropped from the analysis generated similar results as the primary analysis (Supporting Information: Figures 9A to 11B).

## 4 | DISCUSSION

Clinical care is complex, often presents itself as a chaotic mass of data without clear structures and regularity^50^ making use of formal models indispensable.^16,19^ However, it is not clear that decision-making based on the use of models is superior to usual clinical practice, which is typically based on the physicians’ experiential and intuitive reasoning.^3,51–53^

To our knowledge, we report the first study demonstrating that evidence-based guidelines—when delivered via FFT decision tree—can improve some outcomes (hospital stay and admission to ICU) although not other outcomes such as death, VTE or major bleeding. By explicitly and transparently translating key elements of importance for making decisions into FFT, we also respond to the critique that guidelines have inherent ‘integration and black-box’ operation problems.^54^ We have hypothesised that better compliance that standardised, algorithmically delivered intervention assure will result in improved outcomes more directly related to the interventions, but we observed effects only on the LOS10d and ICU admission but not on VTE, death or bleeding.

The explanation for these findings may relate to the lack of power-unlike high VTE and bleeding (and death rates) observed in many COVID-19 studies,^21^ in our analysis we encountered very low rates for all primary outcomes (2%–3% for death rates, <1% for VTE, and about 1.5% for major bleed), similar to those observed in some randomised trials ^26–33^ but typically not in observational studies.^21^ On other hand, the event rate for LOD10d and the ICU admission was much higher, about 10%–13% and 25%–30%, respectively. It may be that most practitioners are well versed in anticoagulation, and that algorithmically driven management would unlikely impact outcomes when the intervention targets these well-known drugs. However, the quality problems with hospital VTE prophylaxis are well documented^55,56^ making it difficult to believe that standardised, evidence-based protocols would not be useful. Indeed, sheer knowledge that one adheres to the state-of-the-art decision algorithm may provide necessary confidence to discharge, or not admit a patient to the ICU where default action would be to act otherwise.

Does delivery of prophylactic anticoagulation via FFT-algorithm provide support for the ASH^25^ and ISTH^35^ guidelines recommending antithrombotic treatment with LMWH at prophylactic rather than therapeutic-intensity doses in hospitalised patients with COVID-19 at low risk to progression to critical diseases? In populations with the very low VTE, death and bleeding rates we observed, this is likely the case particularly when this regimen may improve important hospital outcomes such as the length of stay and averting admission to the ICU.

The main limitation of our study is that we employed an observational (retrospective analysis) rather than a randomised design that would have allowed drawing stronger inferences regarding causal attribution of outcomes to the intervention. In addition, our analysis applies to the population at very low risk for key primary outcomes. Nevertheless, success rate in enroling all (over 4300) consecutive patients hospitalised with COVD-19, use of the state-of-the-art advanced, multifaceted statistical methods, and extensive sensitivity analyses that confirmed the robustness of the findings indicated high validity of the presented results.

## 5 | CONCLUSIONS

By testing effect of decision-making via algorithmically delivered FFT, we showed for the first time that evidence-based guidelines improve clinical outcomes that are not rare. A randomised trial to further test comparative effectiveness of FFT versus usual care in common conditions would open the avenue for more decisive assessment if algorithmically driven decision-making can outperform usual, pattern-recognition, intuitive clinical reasoning.^50–52^

## Supplementary Material

Supplements

## Figures and Tables

**TABLE 1:** Baseline characteristics of the covariates considered for the analysis for decision-making that either followed the pathway (FFT-clinical algorithm) or not (nonpathway)

Total Continuous variables	Non-pathway (treatment off FFT algorithm) 835						Pathway (treatment per FFT algorithm) 948						
	Mean	Med	Min	Max	SD	*N*	Mean	Med	Min	Max	SD	*N*	*P*

BMI	31.65 30.5	18.7	66.8	8.03	743	34.17	32	18.8		69.3	9.25	946	<0.01
CCI	2.43 2	0	14	2.34	835	2.12	2	0		14	2.07	948	0.02
Age (years)	59.07 60	17	121	18.41	835	59.22	60	13		106	16.97	948	0.93
Admission ALT	37.2	27	1	344	36.82	815	45.91	32	1	1068	59.48	943	<0.01
Admission ANC	5.49 4.8	0.8	48.89	3.52	809	5.25	4.7	0.3		22.63	2.77	933	0.52
Admission AST	45.53	34	8	452	43.64	815	53.42	38	7	1172	69.17	943	<0.01
Admission BNP	254.9	35.5	1	10580	901.58	210	84	18	1	2036	198	288	<0.01
Admission BUN	19.13 14	3	129	15.21	828	17.44	14	2		136	13.08	947	0.03
Admission CK	312.28	130	6	6945	652.8	318	318.42	130	13	5924	589.13	632	0.40
GFR	73.86 75.95	4.11	138.67	29.64	835	75.17	78.53	6.43		140.69	28.07	948	0.34
Admission LDH	355.49	322	101	2365	181.65	513	380.01	352	102	6577	287.84	633	<0.01
Admission Albumin	3.48 3.5	1.2	4.9	0.53	815	3.44	3.5	1.3		5	0.47	943	0.05
Admission Calcium	8.88 8.8	6.1	11.6	0.64	828	8.75	8.7	6.2		12.4	0.55	947	<0.01
ddimer	1.76 0.83	0.01	35.59	3.86	567	1.22	0.71	0.02		23.17	1.86	700	<0.01
Admission Platelet	231.61	212	55	800	97.39	834	221.53	207	42	759	84.55	948	0.05
**Categorical variables**	**Number yes**			**%**			**Number yes**			**%**			
Cultures_Positive	31			3.71			20			2.11			0.0466
ID_CONSULT	3			0.36			5			0.53			0.7303
Immunocompromised	37			4.43			28			2.95			0.1012
Pregnant	36			4.31			10			1.05			<0.01
Sepsis	40			4.79			21			2.22			<0.01
Race													<0.01
White-NH	239			28.62			171			18.04			
White-Hisp	76			9.10			117			12.34			
Other	230			27.54			329			34.70			
Black	290			34.73			331			34.92			
Sex													0.9621
Female	429			51.38			489			51.58			
Male	406			48.62			459			48.42			
**Treatment**	**Number yes**			**%**			**Number yes**			**%**			
Oxygen_Orders	543			65.03			788			83.12			<0.01
Supplemental_O2	604			72.34			732			77.22			0.0186
Antibiotics	448			53.65			327			34.49			<0.01
Antivirals	379			45.39			468			49.37			0.0964
Remdesivir	359			42.99			462			48.73			0.0173
Steroids	498			59.64			594			62.66			0.2054

Note: See Figure 2 for details of treatment with anticoagulants.

Abbreviations: ALT, alanine transaminase; ANC, absolute neutrophil count; AST, aspartate aminotransferase; BMI, body mass index; BNP, B-type natriuretic peptide; BUN, blood urea nitrogen; CCI, charlson comorbidity index; CK, creatine kinase; GFR, glomerular filtration rate; LDH, lactate dehydrogenase.

## Data Availability

The data that support the findings of this study are available from the corresponding author upon reasonable request.
