# Supplementary material for: Evaluation of a fast-and-frugal clinical decision algorithm (‘pathways’) on clinical outcomes in hospitalised patients with COVID-19 treated with anticoagulants
Source: J Eval Clin Pract. Author manuscript; Available in PMC 2023 Feb 1. (PMC9840687; doi:10.1111/jep.13780)
Supplement: Supplements [file NIHMS1851662-supplement-Supplements.pdf]

Appendix 1

[Table 1 in the main manuscript shows the baseline characteristics of the covariates used in the analysis. Most variables were not balanced making the analysis based on unadjusted comparison potentially biased.]

App Fig 1a. Comparison of effect of FFT-algorithm vs. management off the algorithm on a primary outcome: **probability of death**. *Left figure*: unadjusted analysis; *right figure*: adjusted analysis. While unadjusted analysis indicates statistically significant effect in favor of using FFT-algorithm, no such effect was observed in the adjusted analysis.

App Fig 1b. Diagnostics for overlap assumption and for balance in the covariate distribution between treated (on FFT-algorithm) and untreated (off FFT-algorithm) for primary outcome**: probability of death**. *Left figure* shows no violation of overlap assumption (which requires an overlap between the treatment and control to meet requirement of exchangeability with respect to all covariates included in the model); *right figure* shows standardized differences before and after propensity score weighting. As it can be seen all variables retained in the analyses were well balanced with SMD<0.1.

App Fig 2a. Comparison of effect of FFT-algorithm vs. management off the algorithm on a primary outcome: **probability of VTE (venous thromboembolism)**. *Left figure*: unadjusted analysis; *right figure*: adjusted analysis. There is no difference in effects between two management strategies on VTE in either unadjusted or adjusted analysis.

App Fig 2b. Diagnostics for overlap assumption and for balance in the covariate distribution between treated (on FFT-algorithm) and untreated (off FFT-algorithm) for primary outcome**: probability of VTE**. *Left figure* shows no violation of overlap assumption (which requires an overlap between the treatment and control to meet requirement of exchangeability with respect to all covariates included in the model); *right figure* shows standardized differences before and after propensity score weighting. As it can be seen all variables retained in the analyses were well balanced with SMD<0.1.

App Fig 3a. Comparison of effect of FFT-algorithm vs. management off the algorithm on a primary outcome: **probability of major bleeding**. *Left figure*: unadjusted analysis; *right figure*: adjusted analysis. There is no difference in effects between two management strategies on major bleeding in either unadjusted or adjusted analysis.

App Fig 3b. Diagnostics for overlap assumption and for balance in the covariate distribution between treated (on FFT-algorithm) and untreated (off FFT-algorithm) for primary outcome**: probability of major bleeding**. *Left figure* shows no violation of overlap assumption (which requires an overlap between the treatment and control to meet requirement of exchangeability with respect to all covariates included in the model); *right figure* shows standardized differences before and after propensity score weighting. As it can be seen all variables retained in the analyses were well balanced with SMD<0.1.

App Fig 4a Comparison of effect of FFT-algorithm vs. management off the algorithm on secondary outcomes: **probability of hospital length of stay >10 days**. Compared with the usual care, FFT-based strategy helped avoid stay in the hospital longer than 10 days by about 3.2% (95% CI: 0.1% to 6.3%) in unadjusted analysis (left figure) and 2.5% (95%CI 0.7 to 4%) in adjusted analysis (*right figure*). This converts into the number of patients needed to be treated

(NNT)=31 (95%CI: 16 to 1,000) in unadjusted analysis [indicating that for every 31 patients (16 to 1,000) managed on FFT algorithm, one avoided staying in hospital longer than 10 days. In adjusted analysis NNT= 40 (95%CI: 23 to 143).

App Fig 4b. Diagnostics for overlap assumption and for balance in the covariate distribution between treated (on FFT-algorithm) and untreated (off FFT-algorithm) for primary outcome**: probability of hospital length of stay >10 days**. *Left figure* shows no violation of overlap assumption (which requires an overlap between the treatment and control to meet requirement of exchangeability with respect to all covariates included in the model); *right figure* shows standardized differences before and after propensity score weighting. As it can be seen all variables retained in the analyses were well balanced with SMD<0.1.

App Fig 5a Comparison of effect of FFT-algorithm vs. management off the algorithm on secondary outcomes: **probability of ICU admission**. Compared with the usual care, FFT-based strategy helped avert admission to intensive-care unit (ICU) by about 8% (95%CI: 4 to 11%) in unadjusted analysis (left figure) and by about 5% (95%CI 2.5 to 8%) in adjusted analysis (*right figure*). This converts into the number of patients needed to be treated to avert one admission to ICU (NNT)=13 (95%CI: 9 to 25) in unadjusted and 19 (95%CI: 13 to 40] in adjusted analysis, respectively.

App Fig 5b. Diagnostics for overlap assumption and for balance in the covariate distribution between treated (on FFT-algorithm) and untreated (off FFT-algorithm) for primary outcome**: probability of ICU admission**. *Left figure* shows no violation of overlap assumption (which requires an overlap between the treatment and control to meet requirement of exchangeability with respect to all covariates included in the model); *right figure* shows standardized differences before and after propensity score weighting. As it can be seen all variables retained in the analyses were well balanced with SMD<0.1.

Sensitivity analyses (SA)

A) missing data (BNP, D-dimer, LDH, CK)dropped

App Fig 6a. Comparison of effect of FFT-algorithm vs. management off the algorithm on a primary outcome: **probability of death**. *Left figure*: unadjusted analysis; *right figure*: adjusted analysis. No meaningful differences from the findings obtained in the main analysis was seen (App1 Fig 1)

App Fig 6b. Diagnostics for overlap assumption and for balance in the covariate distribution between treated (on FFT-algorithm) and untreated (off FFT-algorithm) for primary outcome**: probability of death**. *Left figure* shows no violation of overlap assumption (which requires an overlap between the treatment and control to meet requirement of exchangeability with respect to the all covariates included in the model); *right figure* shows standardized differences before and after propensity score weighting. As it can be seen all variables retained in the analyses were well balanced with SMD<0.1.

App Fig 7a. Comparison of effect of FFT-algorithm vs. management off the algorithm on a primary outcome: **probability of VTE (venous thromboembolism)**. *Left figure*: unadjusted analysis; *right figure*: adjusted analysis. No meaningful differences from the findings obtained in the main analysis was seen (App1 Fig 2)

App Fig 7b. Diagnostics for overlap assumption and for balance in the covariate distribution between treated (on FFT-algorithm) and untreated (off FFT-algorithm) for primary outcome**: probability of VTE**. *Left figure* shows no violation of overlap assumption (which requires an overlap between the treatment and control to meet requirement of exchangeability with respect to all covariates included in the model); *right figure* shows standardized differences before and after propensity score weighting. As it can be seen all variables retained in the analyses were well balanced with SMD<0.1.

App Fig 8a. Comparison of effect of FFT-algorithm vs. management off the algorithm on a primary outcome: **probability of major bleeding**. *Left figure*: unadjusted analysis; *right figure*: adjusted analysis. No meaningful differences from the findings obtained in the main analysis was seen (App1 Fig 3)

App Fig 8b. Diagnostics for overlap assumption and for balance in the covariate distribution between treated (on FFT-algorithm) and untreated (off FFT-algorithm) for primary outcome**: probability of major bleeding**. *Left figure* shows no violation of overlap assumption (which requires an overlap between the treatment and control to meet requirement of exchangeability with respect to all covariates included in the model); *right figure* shows standardized differences before and after propensity score weighting. As it can be seen all variables retained in the analyses were well balanced with SMD<0.1.

B) Dropping the data ("AC switch") with potentially invalid ascertainment of the exposure (i.e., prophylactic vs. therapeutic assignment; "AC switch")

App Fig 9a. Comparison of effect of FFT-algorithm vs. management off the algorithm on a primary outcome: **probability of death**. *Left figure*: unadjusted analysis; *right figure*: adjusted analysis. No meaningful differences from the findings obtained in the main analysis was seen (App1 Fig 1)

App Fig 9b. Diagnostics for overlap assumption and for balance in the covariate distribution between treated (on FFT-algorithm) and untreated (off FFT-algorithm) for primary outcome**: probability of death**. *Left figure* shows no violation of overlap assumption (which requires an overlap between the treatment and control to meet requirement of exchangeability with respect to the all covariates included in the model); *right figure* shows standardized differences before and after propensity score weighting. As it can be seen all variables retained in the analyses were well balanced with SMD<0.1.

App Fig 10a. Comparison of effect of FFT-algorithm vs. management off the algorithm on a primary outcome: **probability of VTE (venous thromboembolism)**. *Left figure*: unadjusted analysis; *right figure*: adjusted analysis. No meaningful differences from the findings obtained in the main analysis was seen (App1 Fig 2)

App Fig 10b. Diagnostics for overlap assumption and for balance in the covariate distribution between treated (on FFT-algorithm) and untreated (off FFT-algorithm) for primary outcome**: probability of VTE**. *Left figure* shows no violation of overlap assumption (which requires an overlap between the treatment and control to meet requirement of exchangeability with respect to all covariates included in the model); *right figure* shows standardized differences before and

after propensity score weighting. As it can be seen all variables retained in the analyses were well balanced with SMD<0.1.

App Fig 11a. Comparison of effect of FFT-algorithm vs. management off the algorithm on a primary outcome: **probability of major bleeding**. *Left figure*: unadjusted analysis; *right figure*: adjusted analysis. No meaningful differences from the findings obtained in the main analysis was seen (App1 Fig 3)

App Fig 11b. Diagnostics for overlap assumption and for balance in the covariate distribution between treated (on FFT-algorithm) and untreated (off FFT-algorithm) for primary outcome**: probability of major bleeding**. *Left figure* shows no violation of overlap assumption (which requires an overlap between the treatment and control to meet requirement of exchangeability with respect to all covariates included in the model); *right figure* shows standardized differences before and after propensity score weighting. As it can be seen all variables retained in the analyses were well balanced with SMD<0.1.

[Similar results were obtained for secondary outcomes]

Effect of FFT algorithm on outcomes: death

Unadjusted analyses

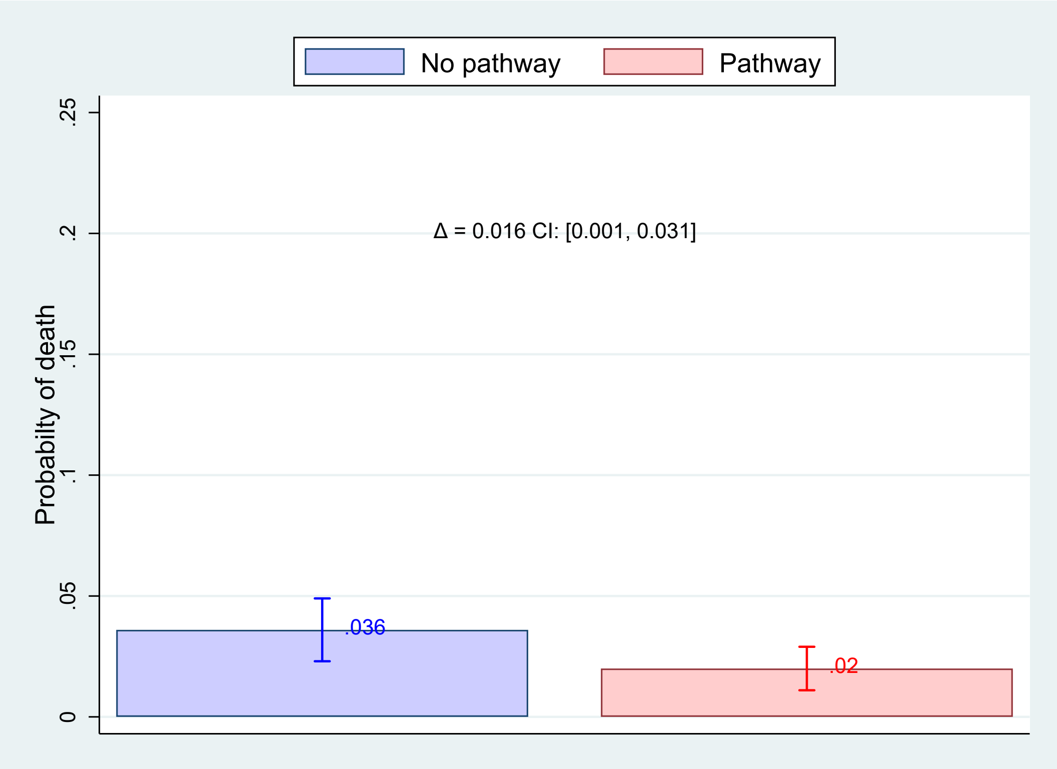

Adjusted analyses

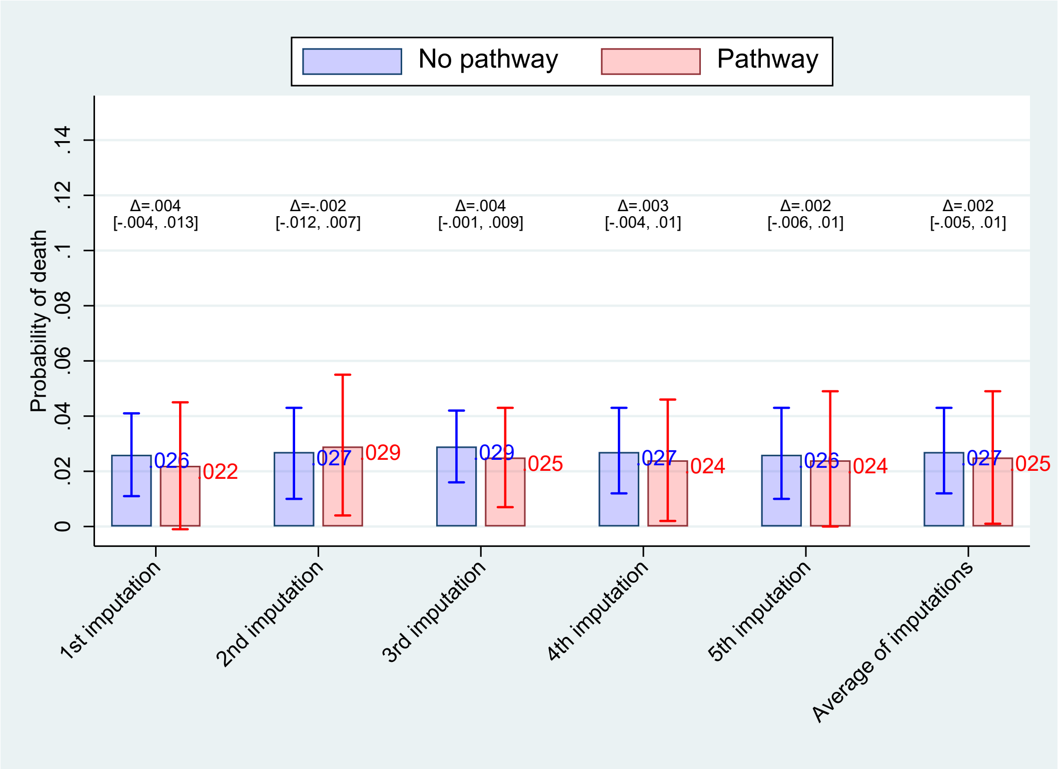

App Fig 1a

Diagnostics for overlap assumption and for balance in the covariate distribution between treated (on FFT-algorithm) and untreated (off FFT-algorithm): death

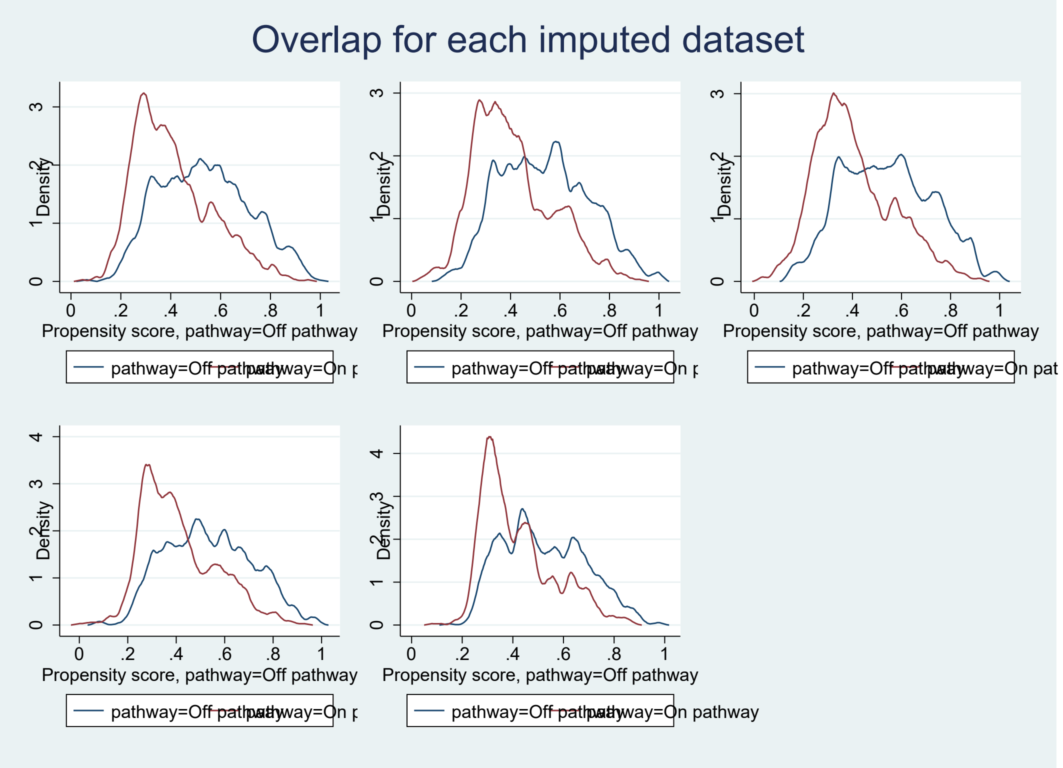

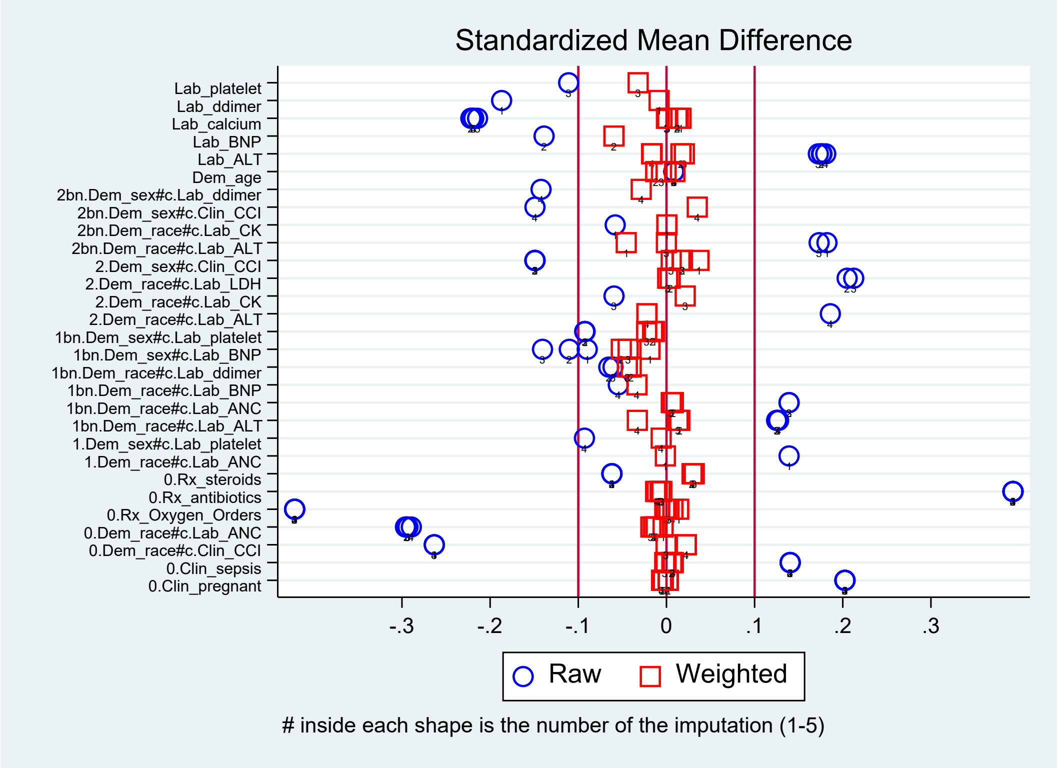

App Fig 1b

Effect of FFT algorithm on outcomes: VTE

Unadjusted

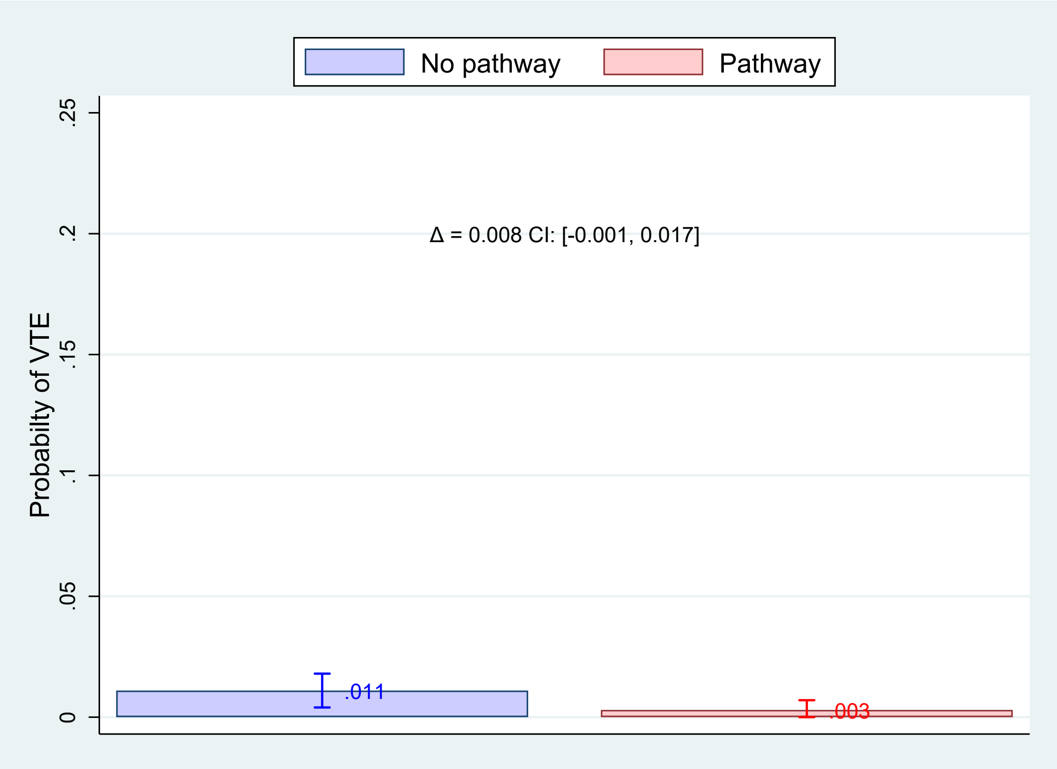

Adjusted

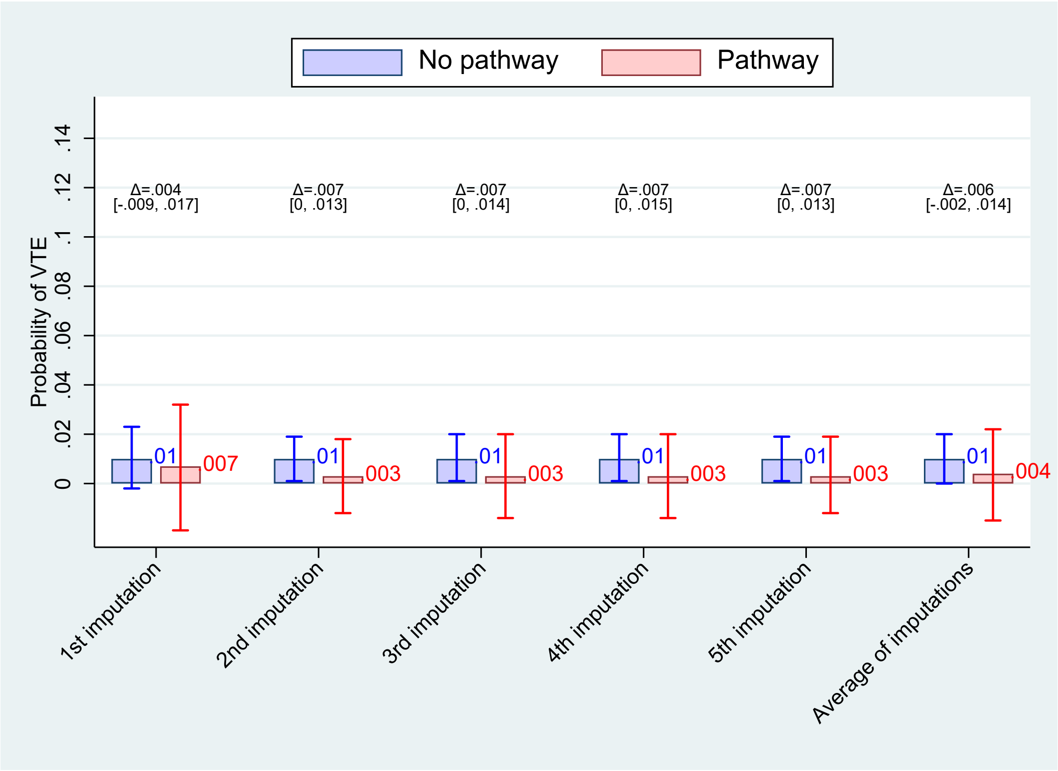

App Fig 2a

Diagnostics for overlap assumption and for balance in the covariate distribution between treated (on FFT-algorithm) and untreated (off FFT-algorithm): VTE

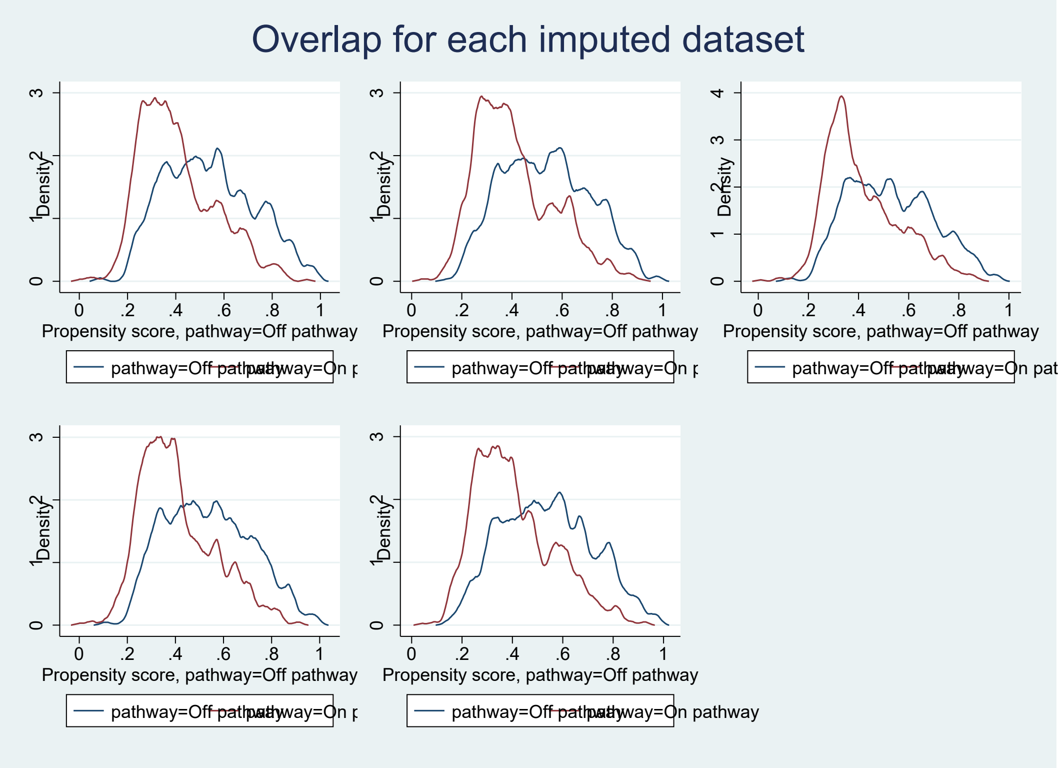

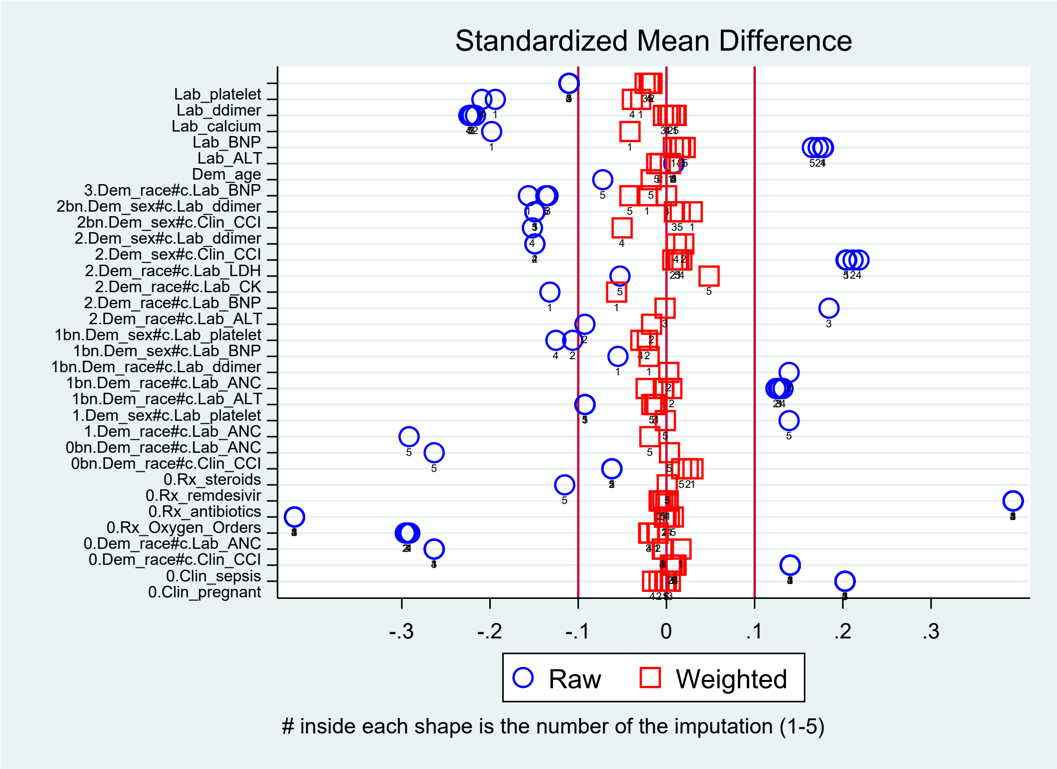

App Fig 2b

Effect of FFT algorithm on outcomes: major bleed

Unadjusted

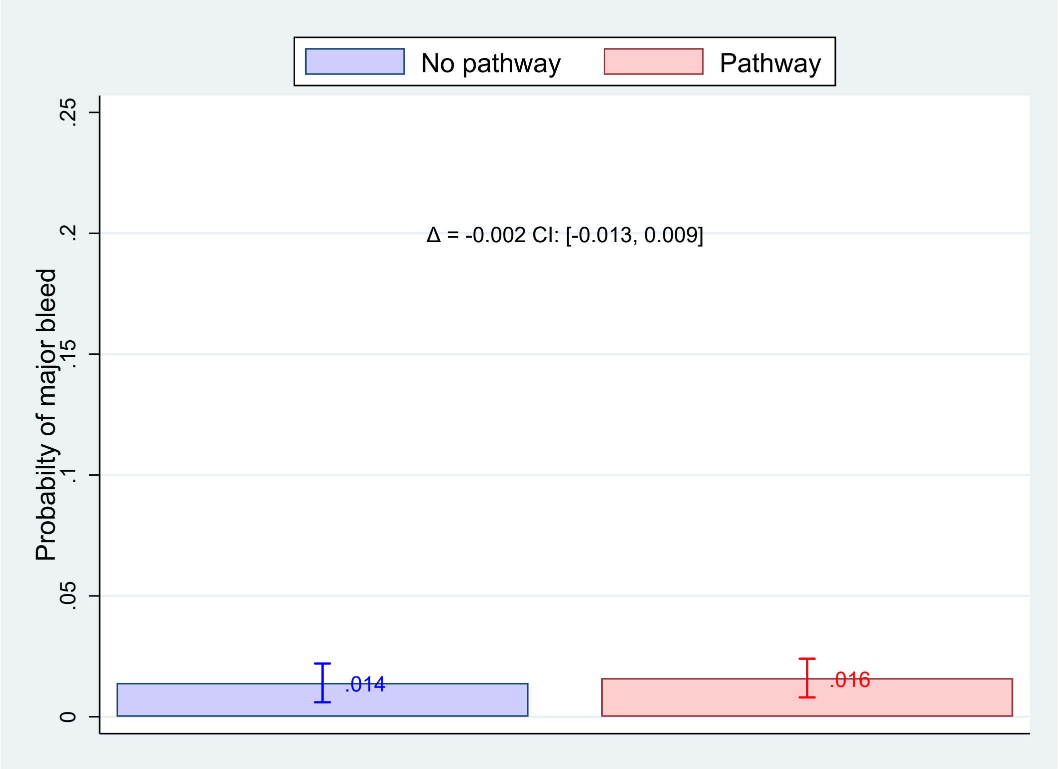

Adjusted

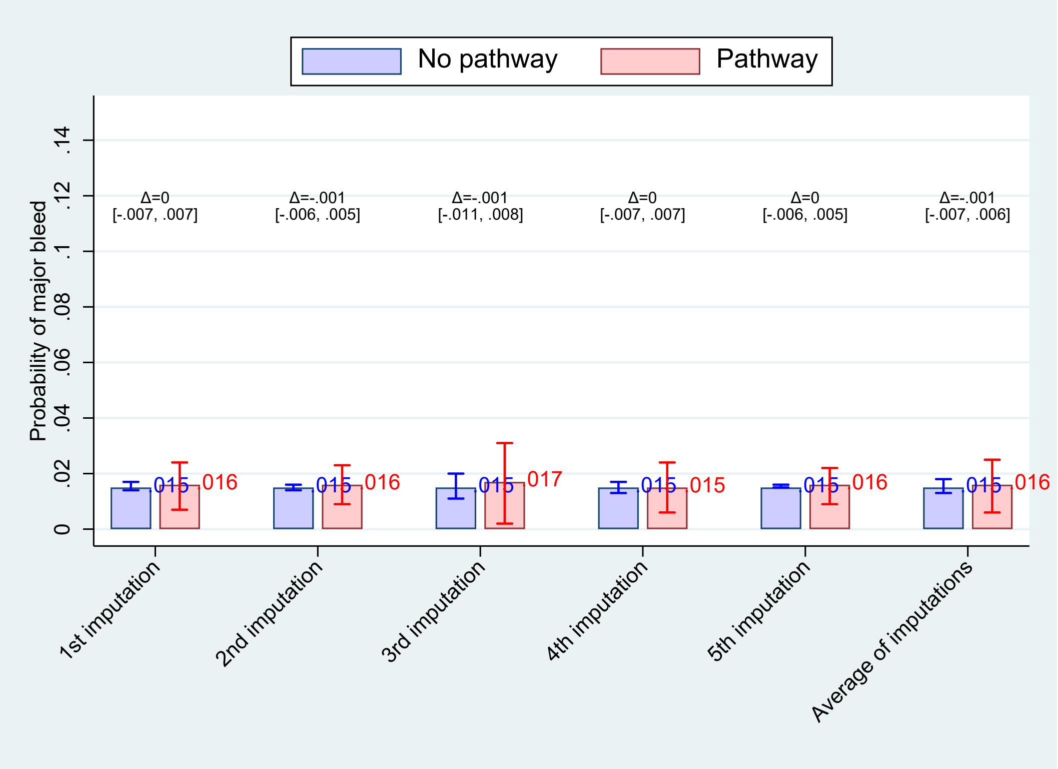

App Fig 3a

Diagnostics for overlap assumption and for balance in the covariate distribution between treated (on FFT-algorithm) and untreated (off FFT-algorithm): major bleed

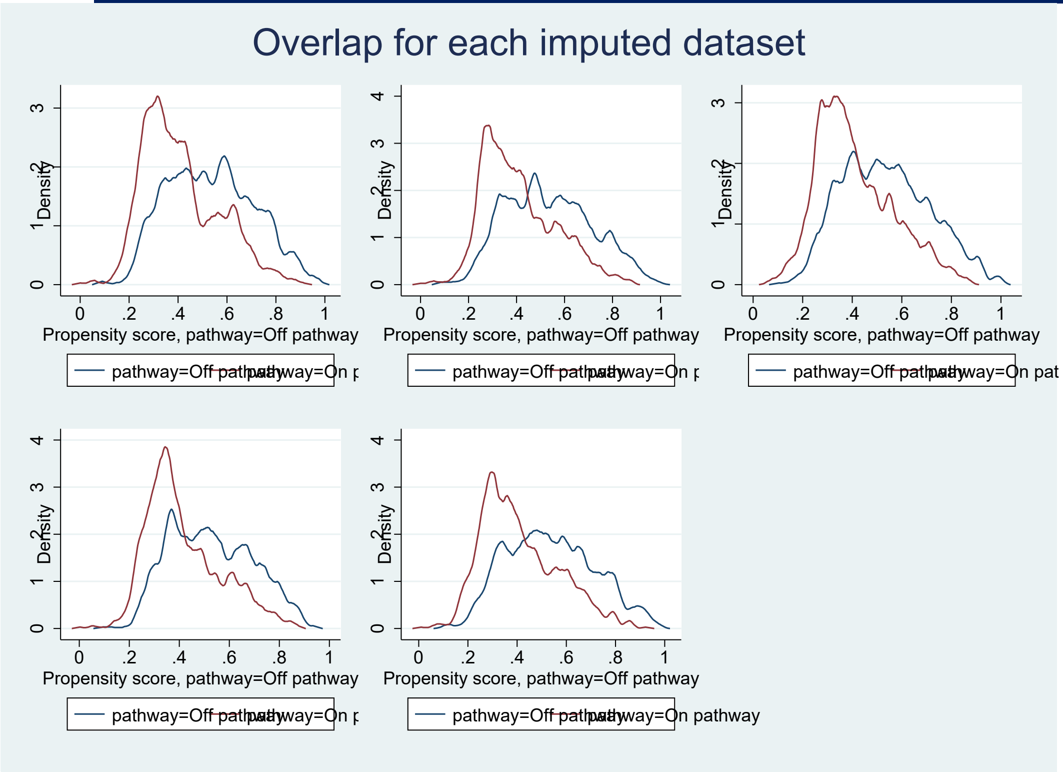

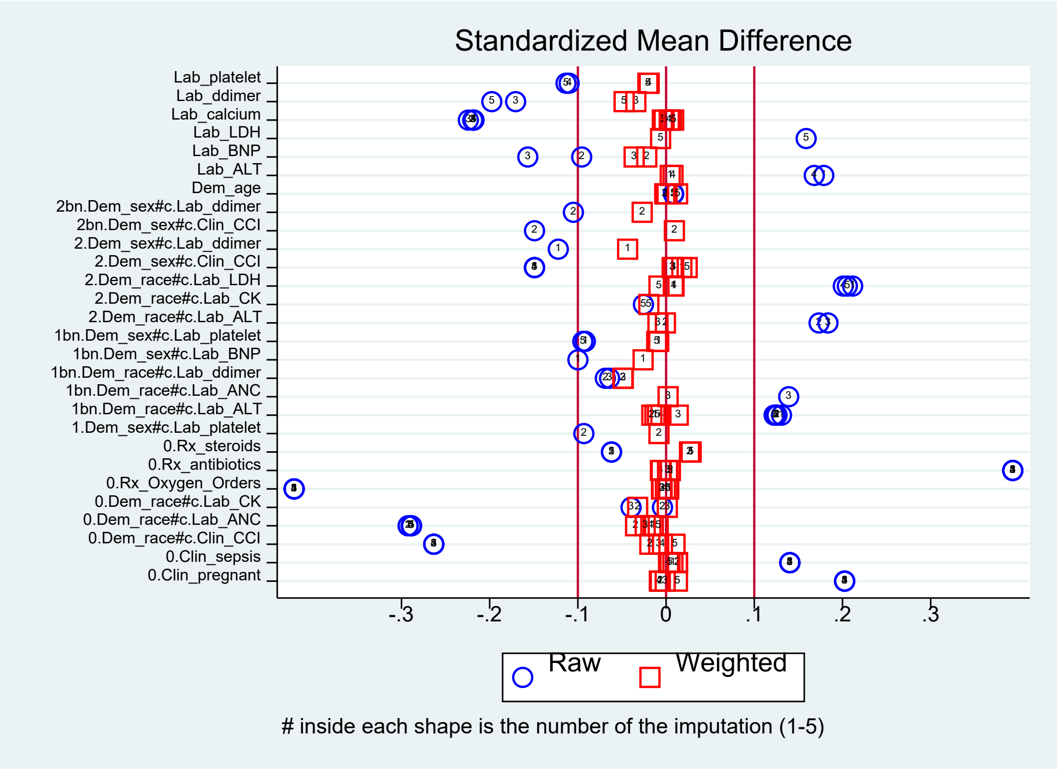

App Fig 3b

Secondary outcomes:LOS (>10 days)

Unadjusted

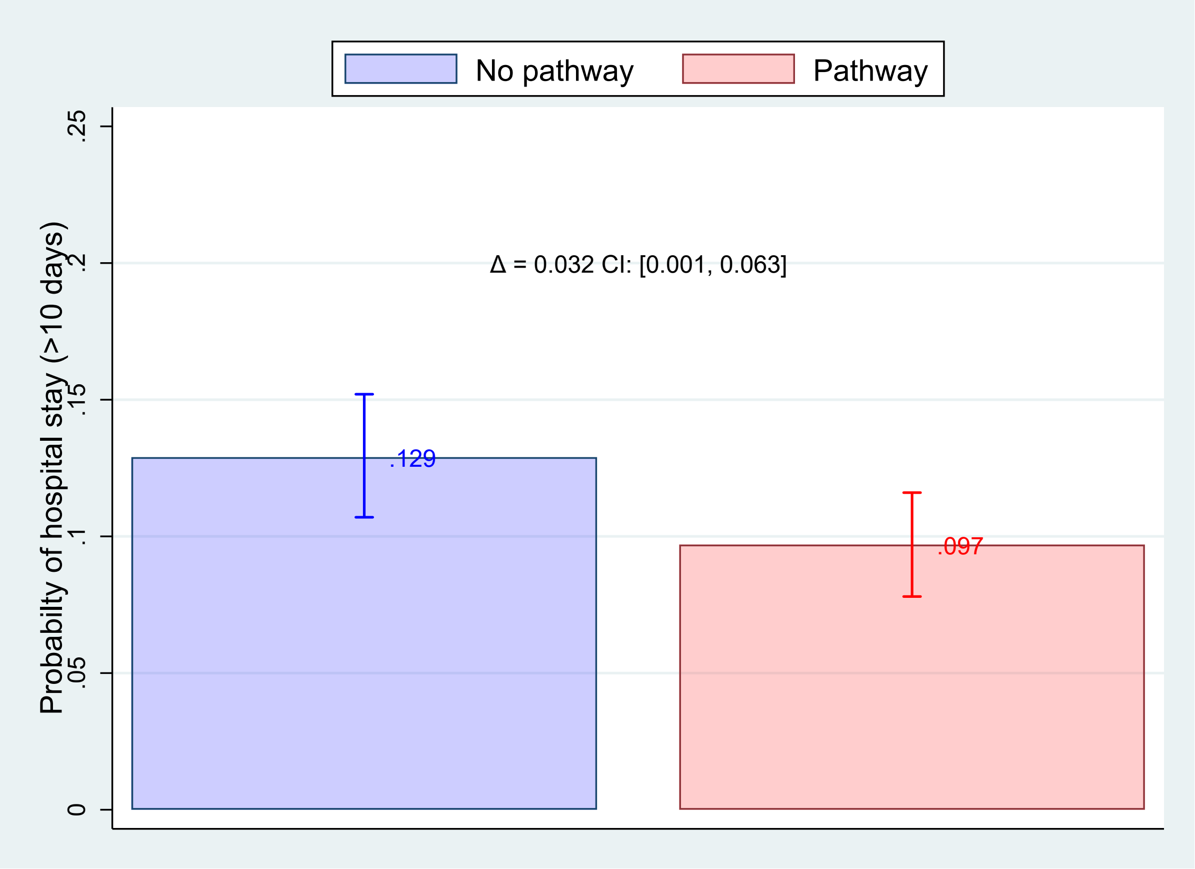

Adjusted

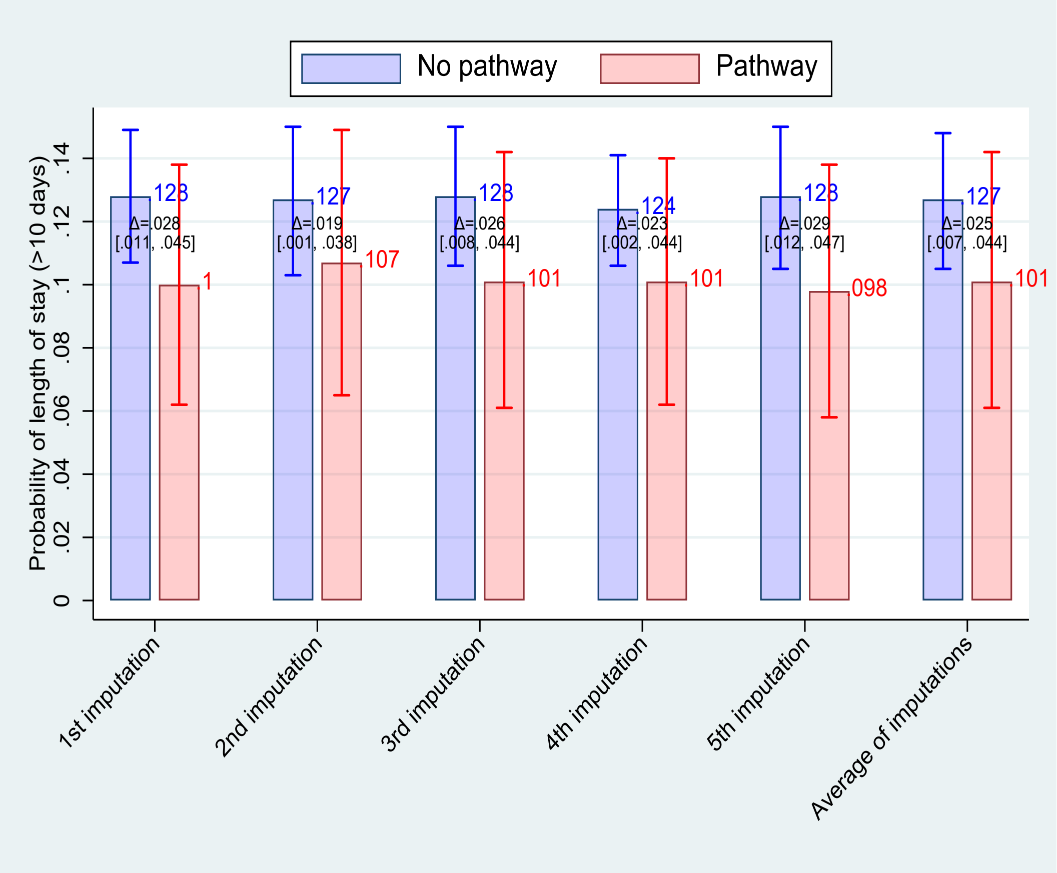

App Fig 4a

Diagnostics for overlap assumption and for balance in the covariate distribution between treated (on FFT-algorithm) and untreated (off FFT-algorithm): LOS (>10 days)

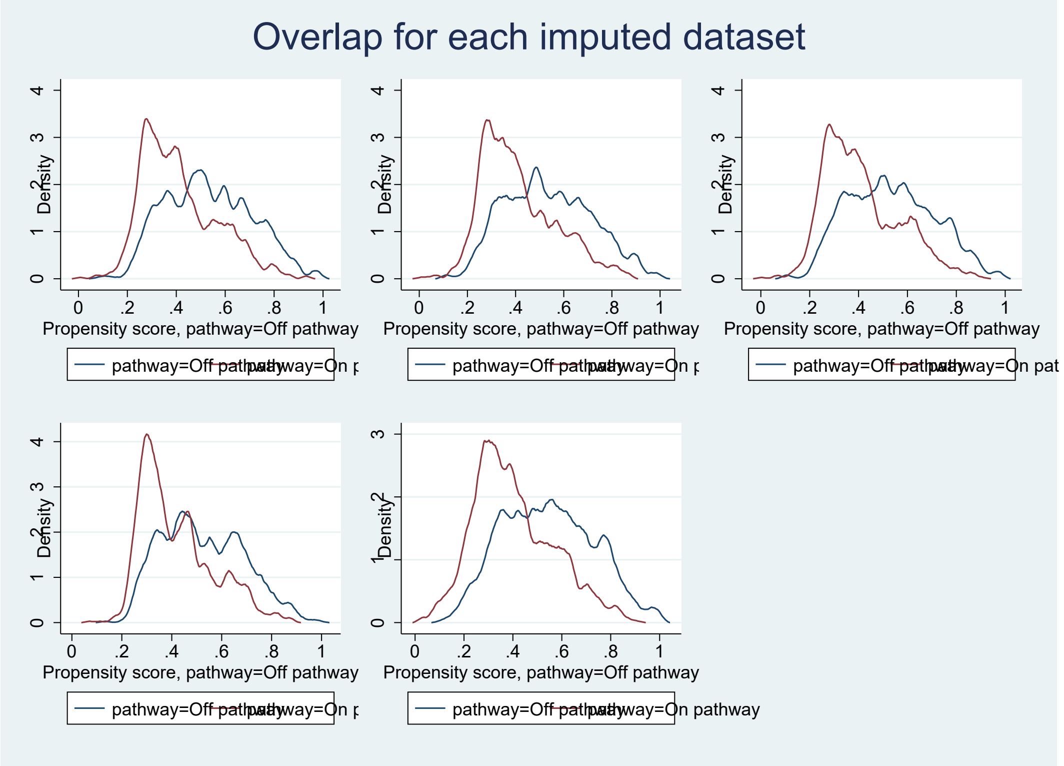

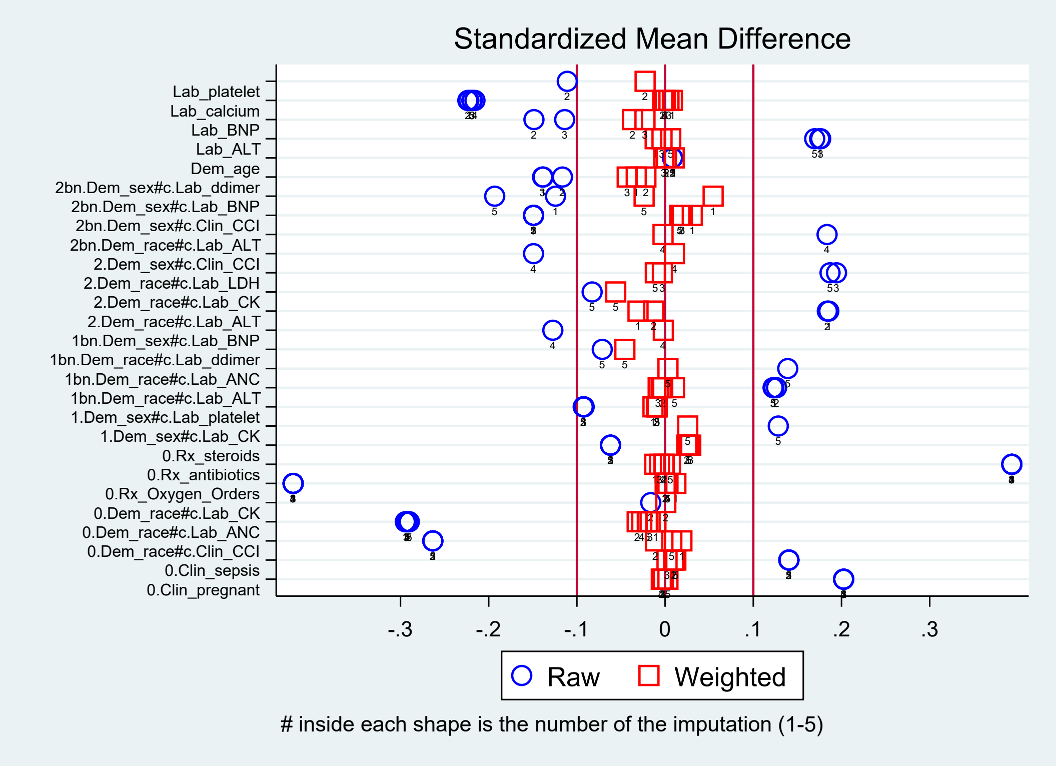

App Fig 4b

Secondary outcomes:ICU admission

Unadjusted

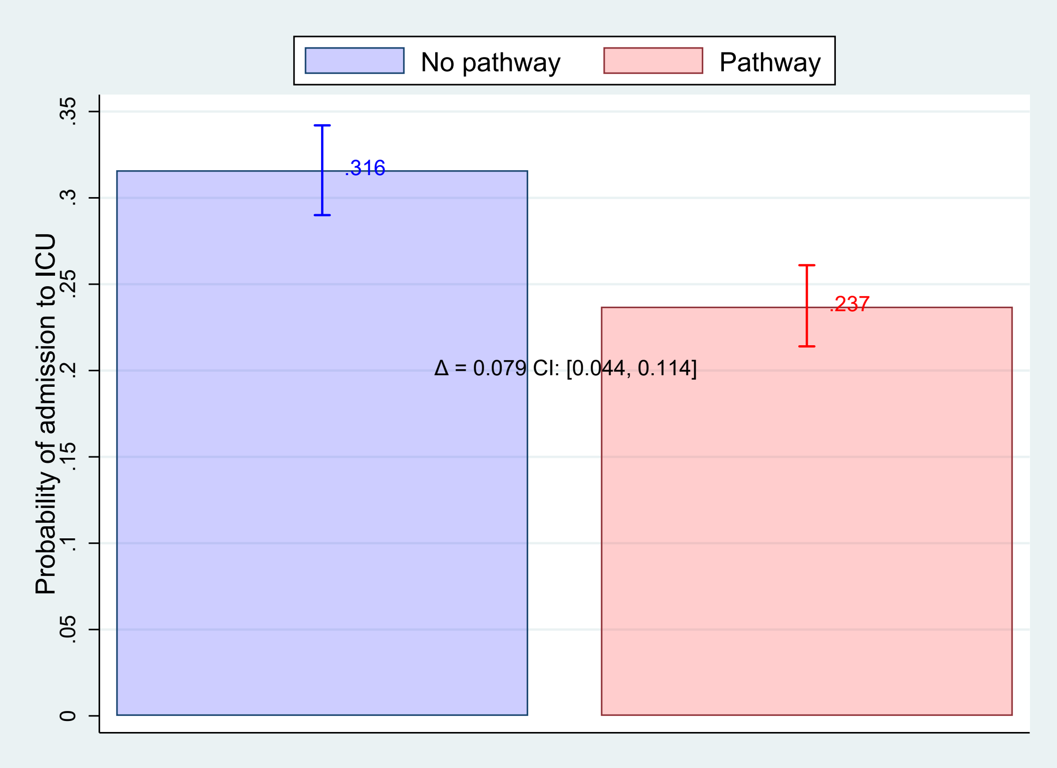

Adjusted

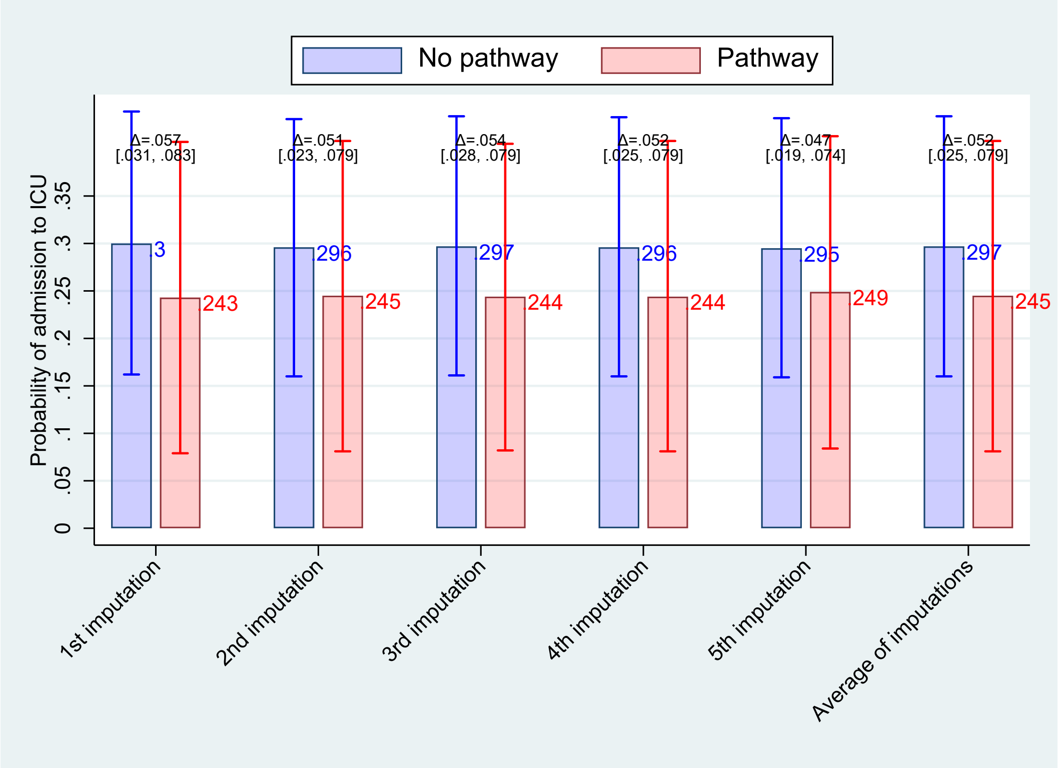

App Fig 5a

Diagnostics for overlap assumption and for balance in the covariate distribution between treated (on FFT-algorithm) and untreated (off FFT-algorithm): ICU admission

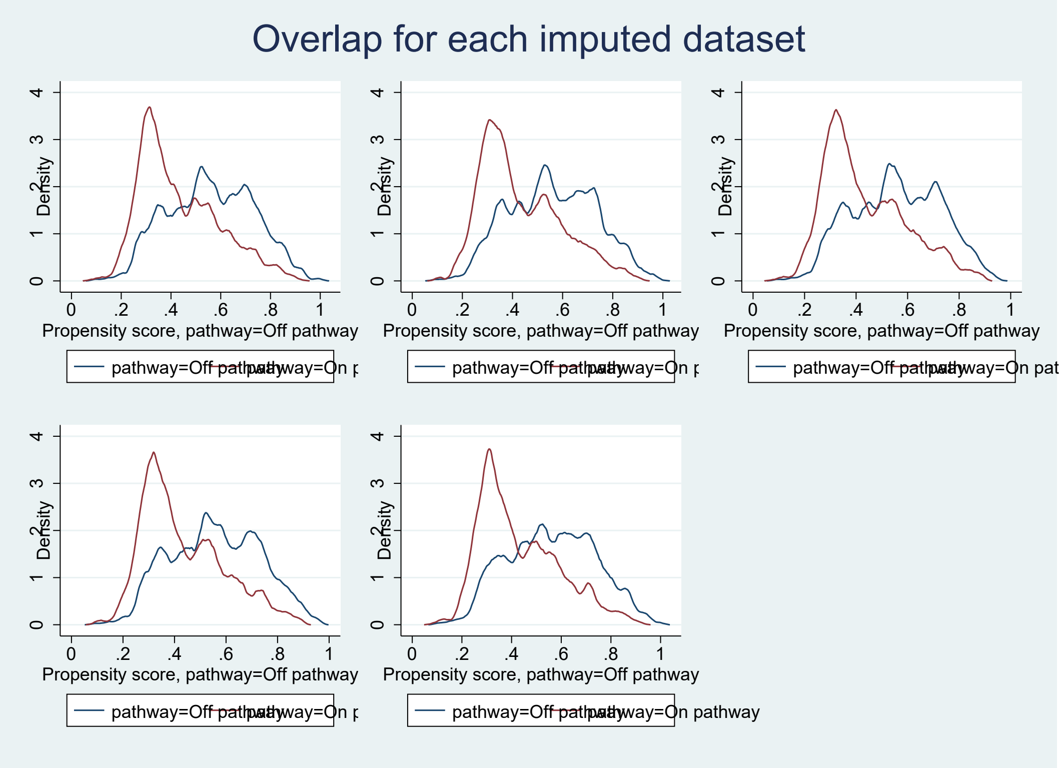

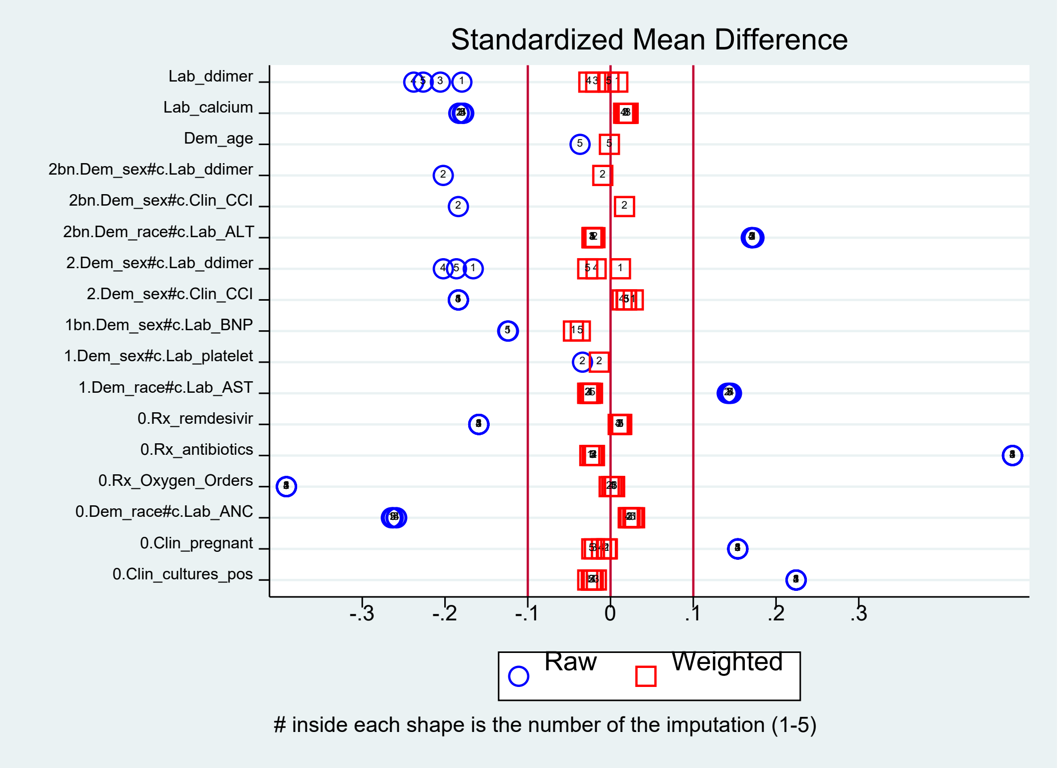

App Fig 5b

Sensitivity analyses (SA):
A) missing data (BNP, D-dimer LDH, CK)dropped

SA- Effect of FFT algorithm on outcomes: death

All data

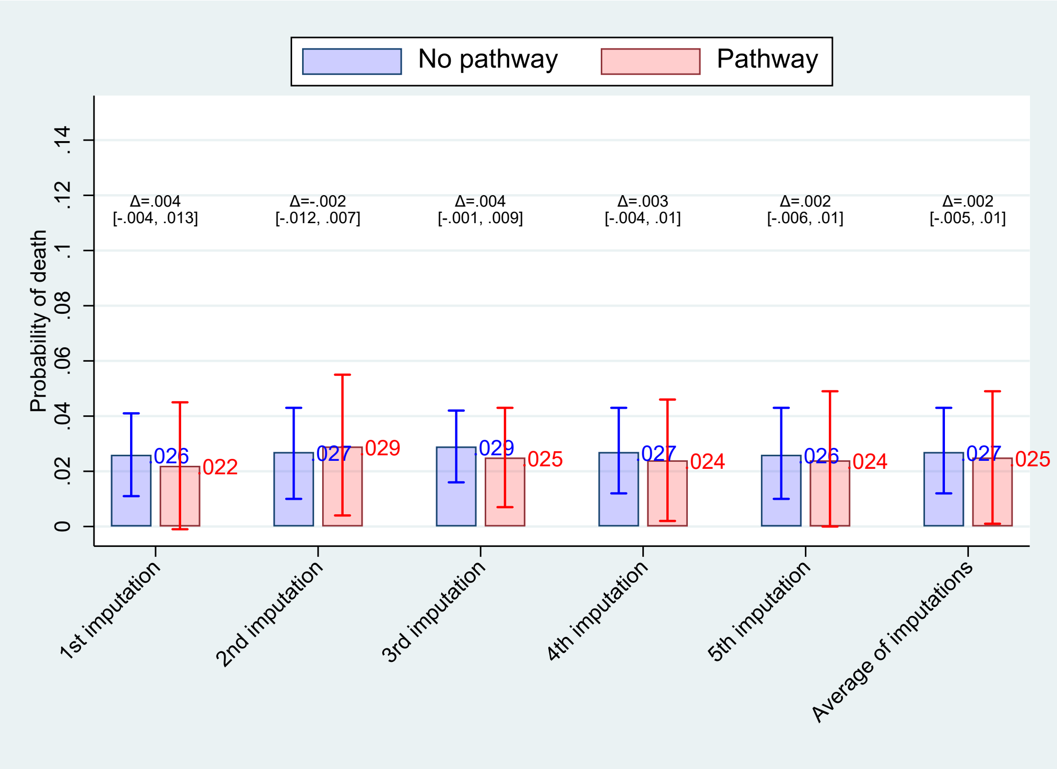

After dropping (BNP, D-dimer, LDH, CK)

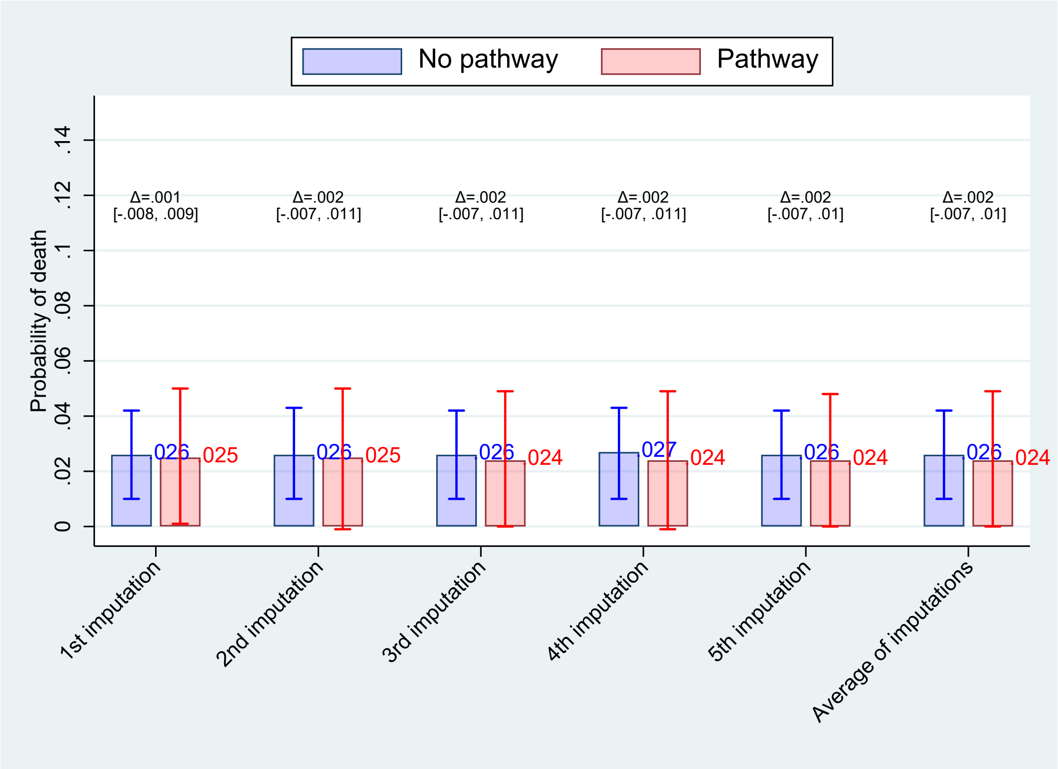

App Fig 6a

SA-Diagnostics for overlap assumption and for balance in the covariate distribution between treated (on FFT-algorithm) and untreated (off FFT-algorithm): death

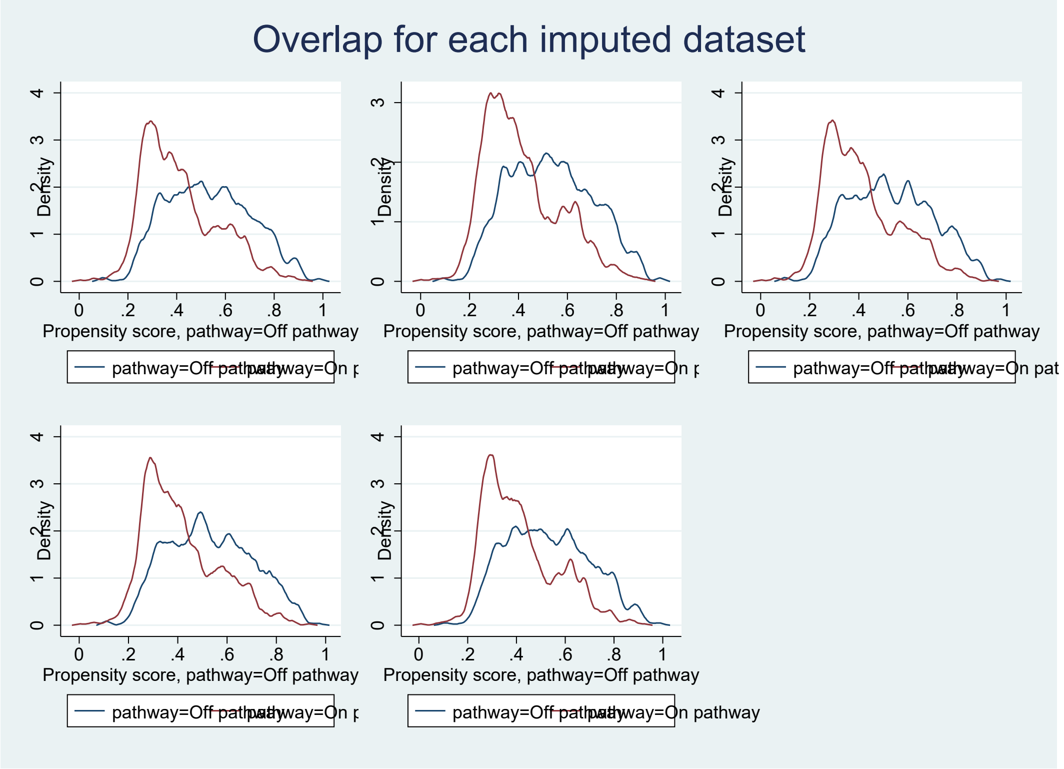

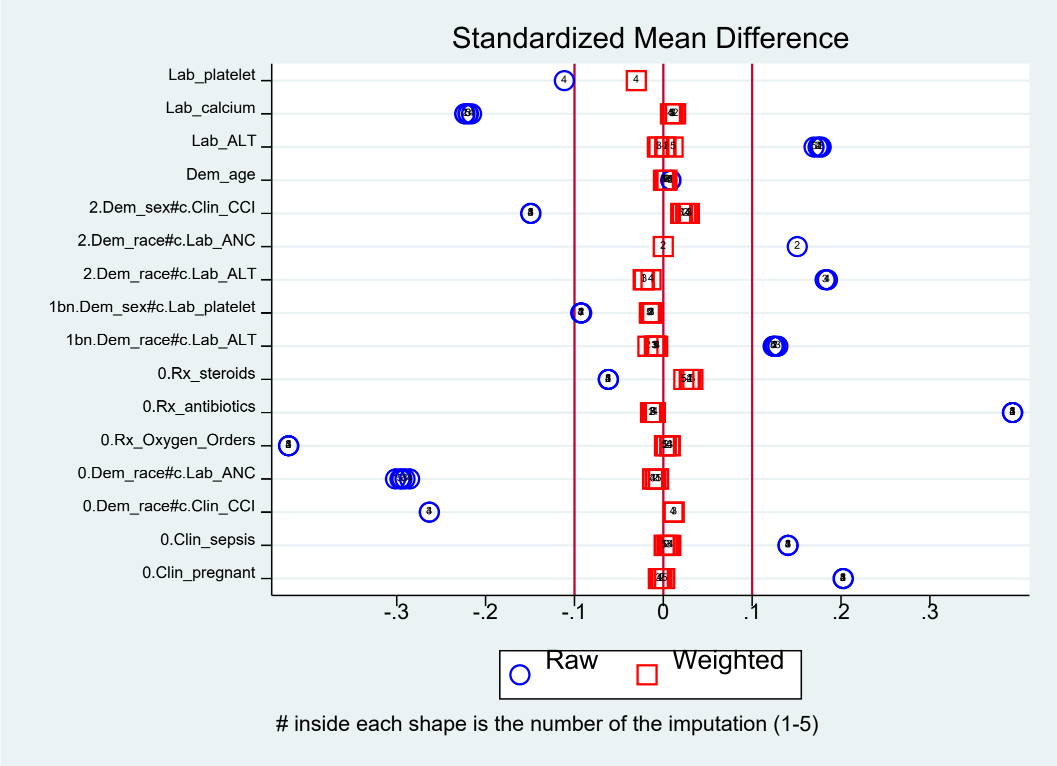

App Fig 6b

SA-Effect of FFT algorithm on outcomes: VTE

All data

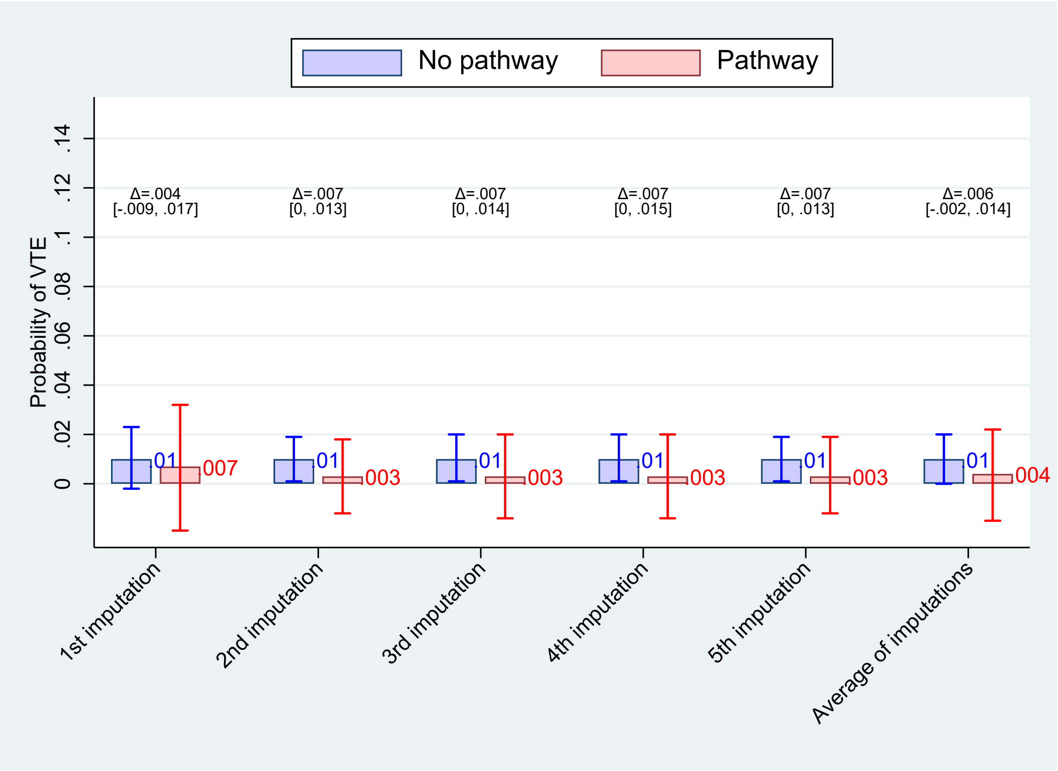

After dropping (BNP, D-dimer, LDH, CK)

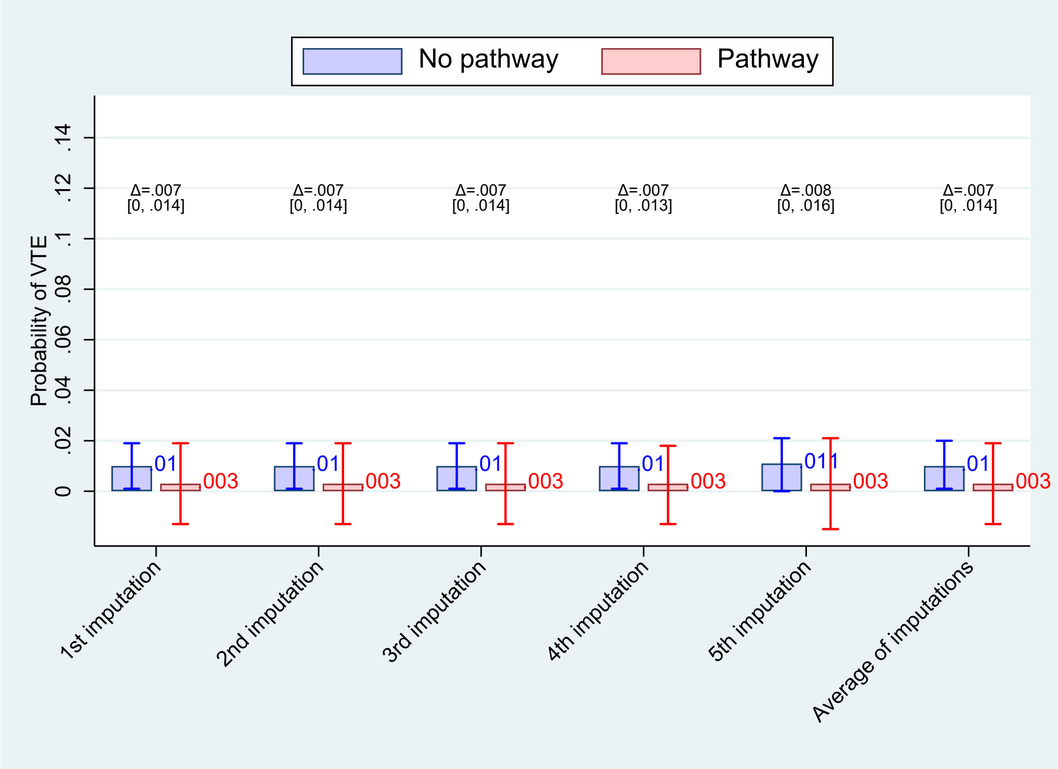

App Fig 7a

SA-Diagnostics for overlap assumption and for balance in the covariate distribution between treated (on FFT-algorithm) and untreated (off FFT-algorithm): VTE

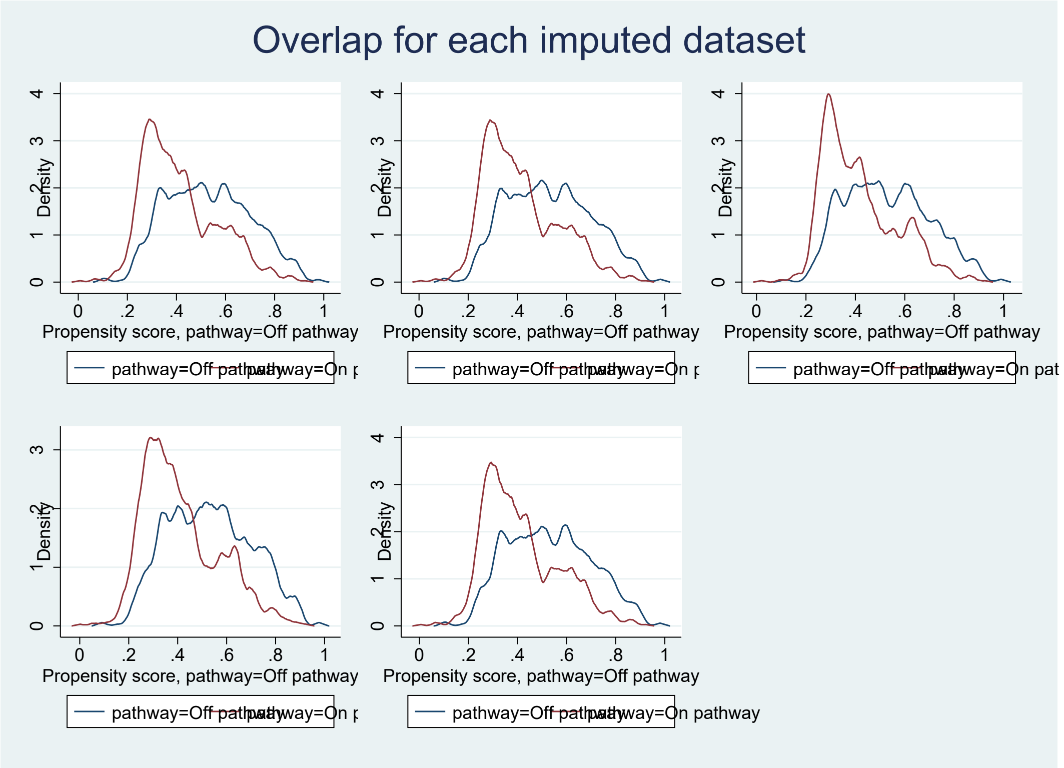

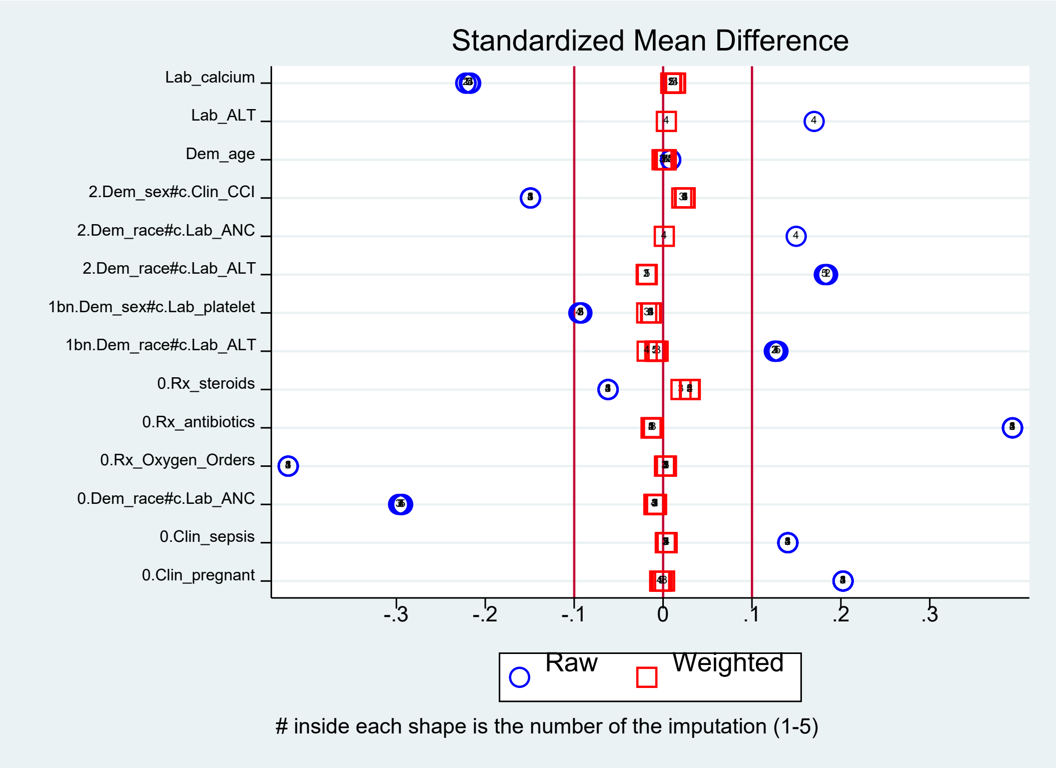

App Fig 7b

SA-Effect of FFT algorithm on outcomes: major bleed

All data

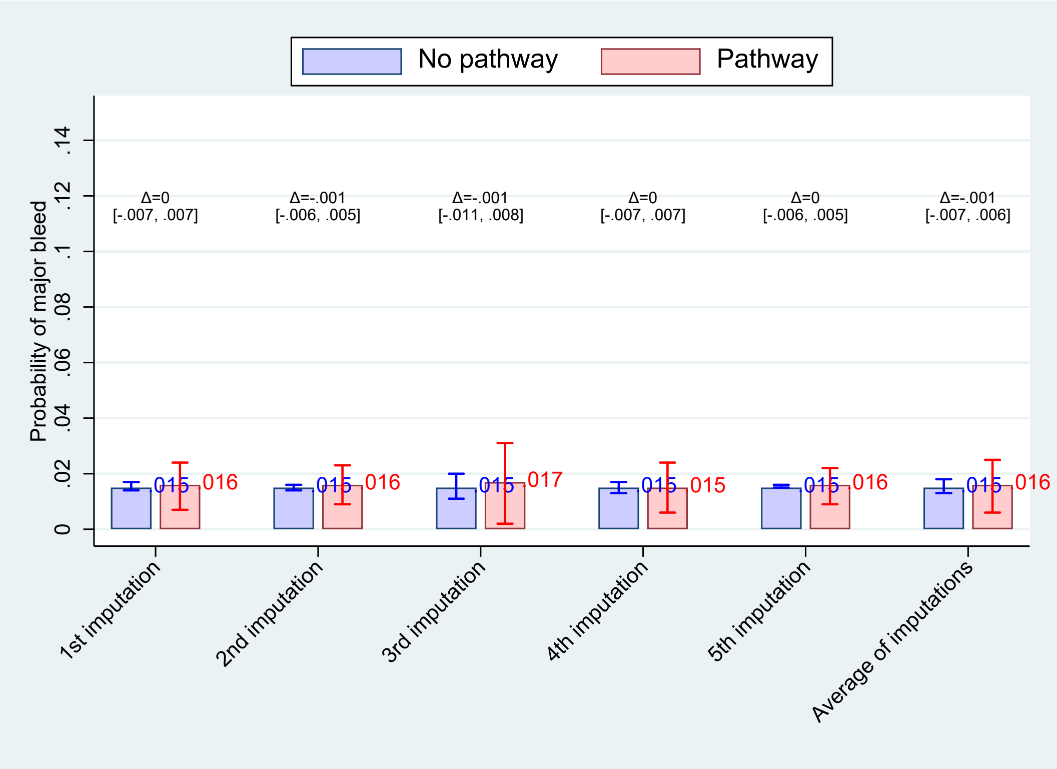

After dropping (BNP, D-dimer, LDH, CK)

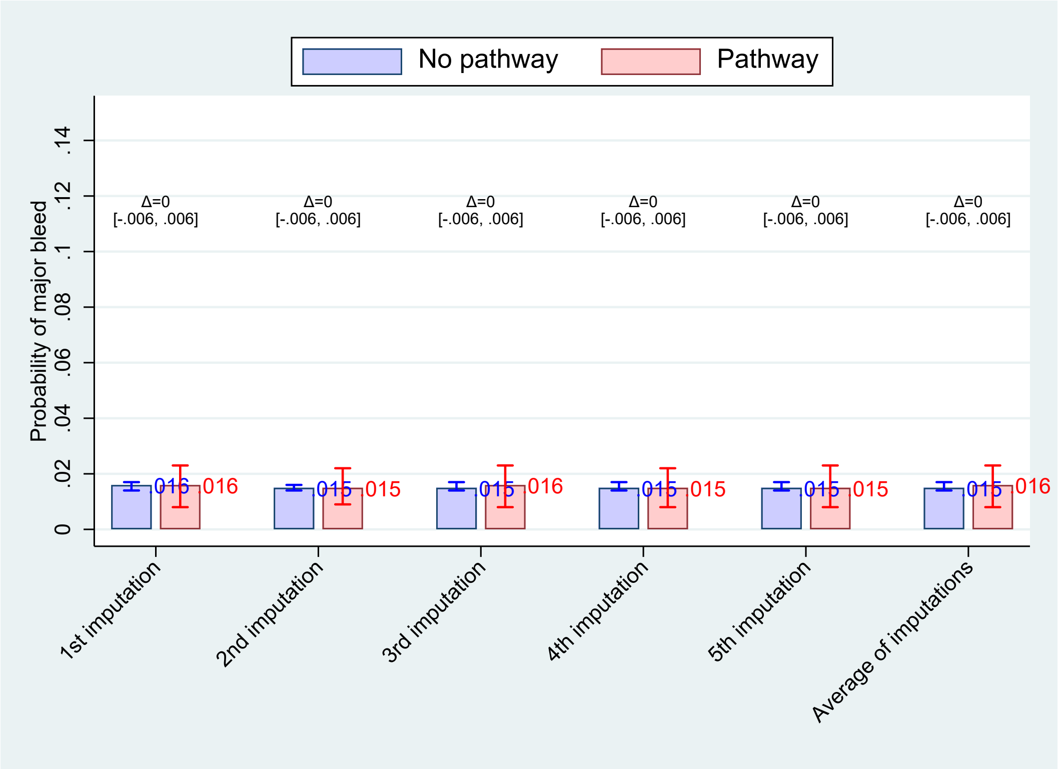

App Fig 8a

SA-Diagnostics for overlap assumption and for balance in the covariate distribution between treated (on FFT-algorithm) and untreated (off FFT-algorithm): major bleed

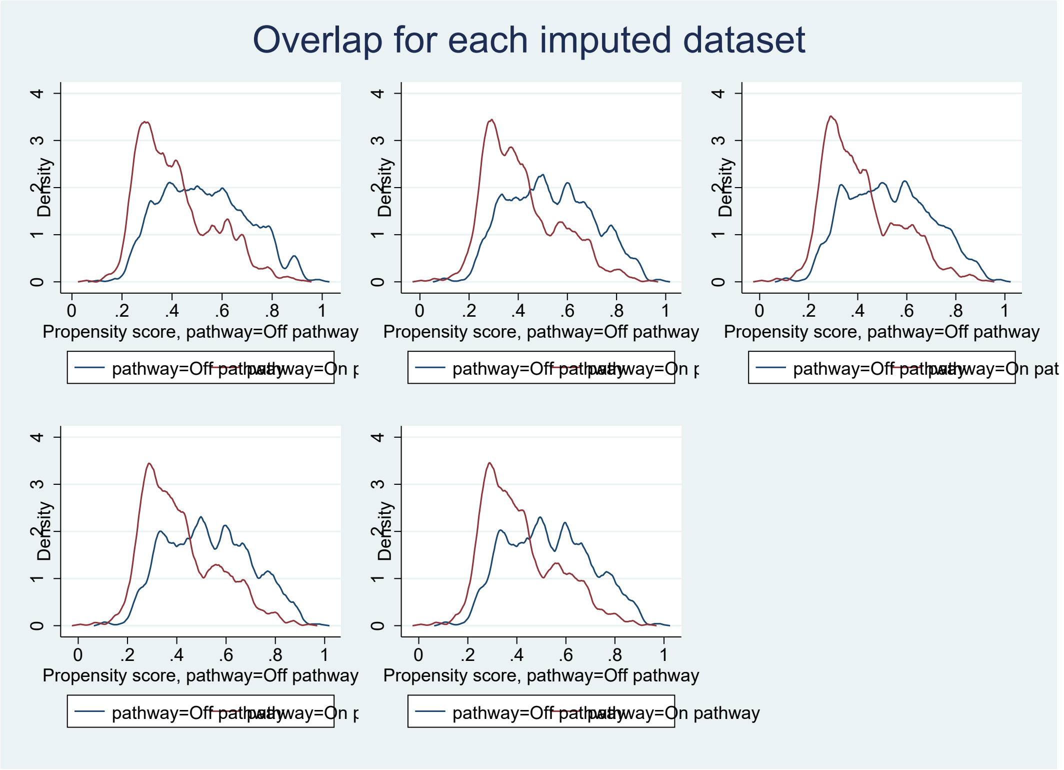

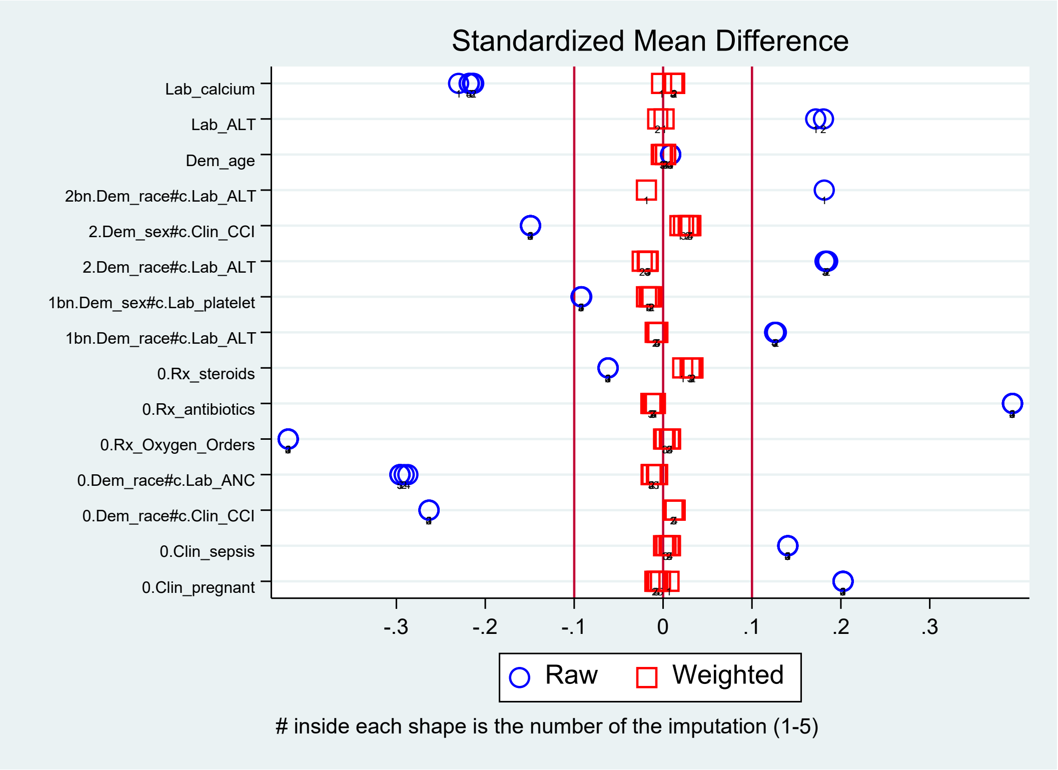

App Fig 8b

Sensitivity analyses:
B) AC switch dropped

SA- Effect of FFT algorithm on outcomes: death

All data

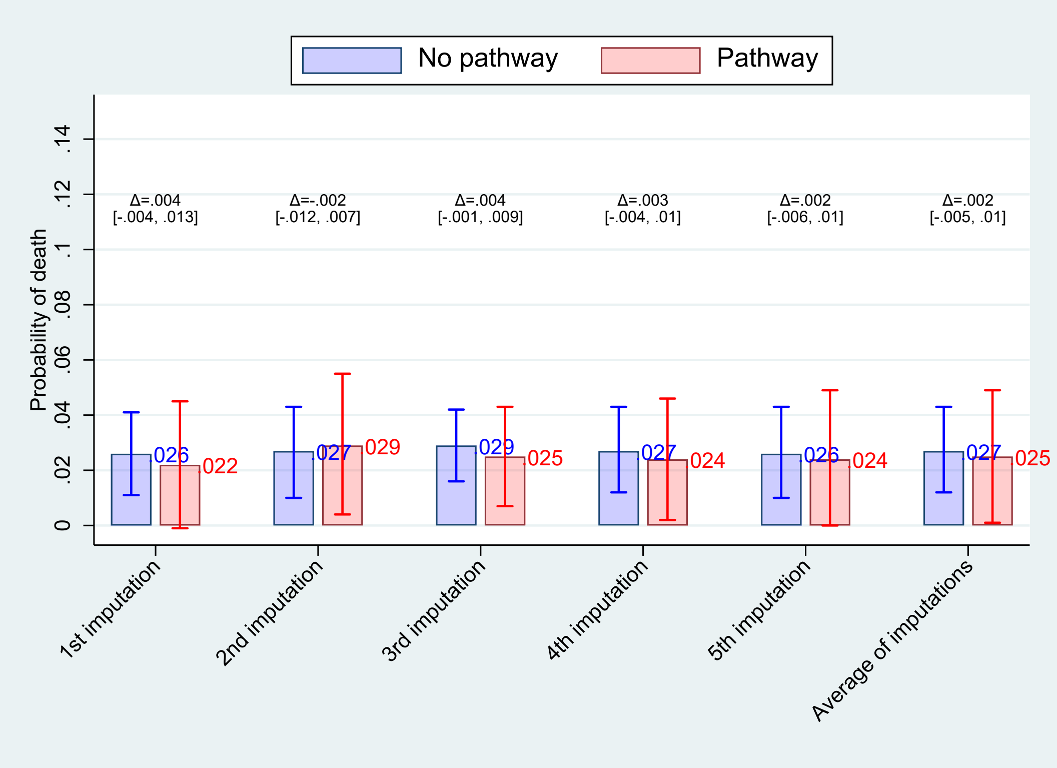

After dropping AC switch

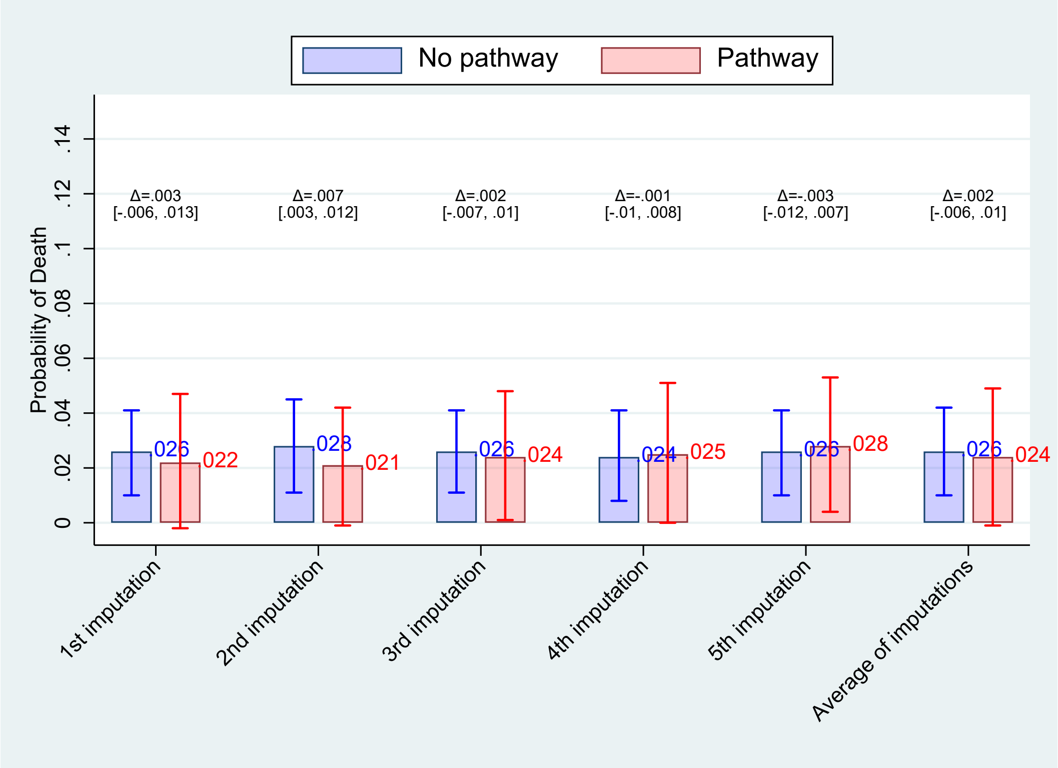

App Fig 9a

SA-Diagnostics for overlap assumption and for balance in the covariate distribution between treated (on FFT-algorithm) and untreated (off FFT-algorithm): death

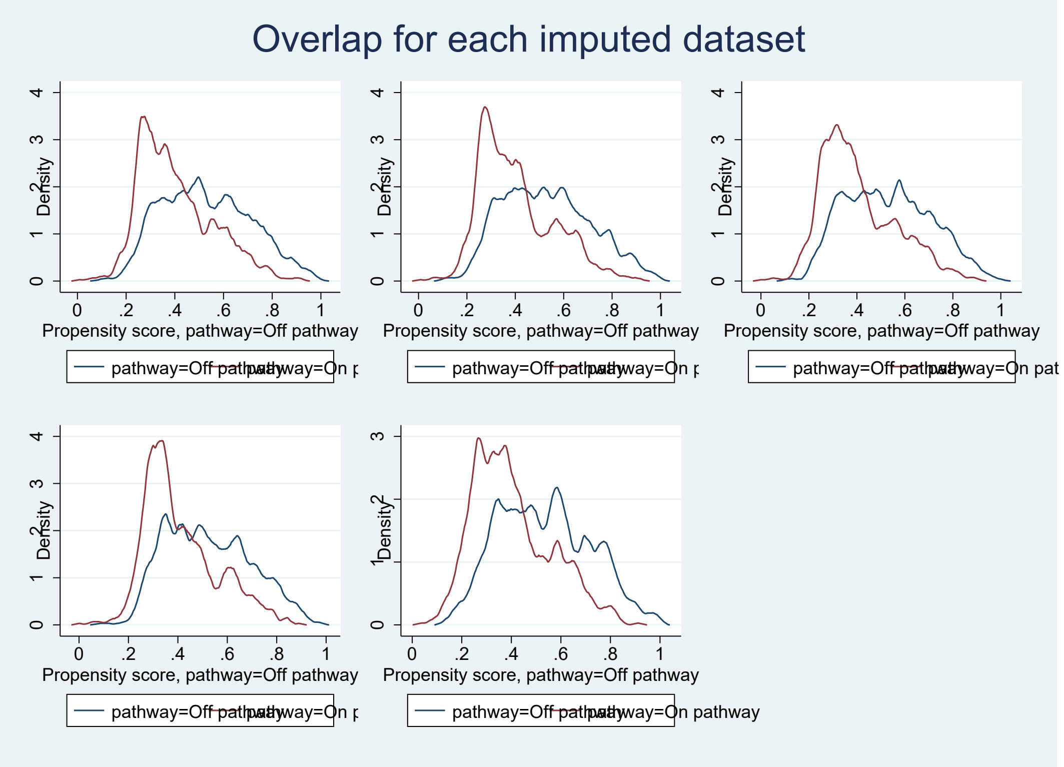

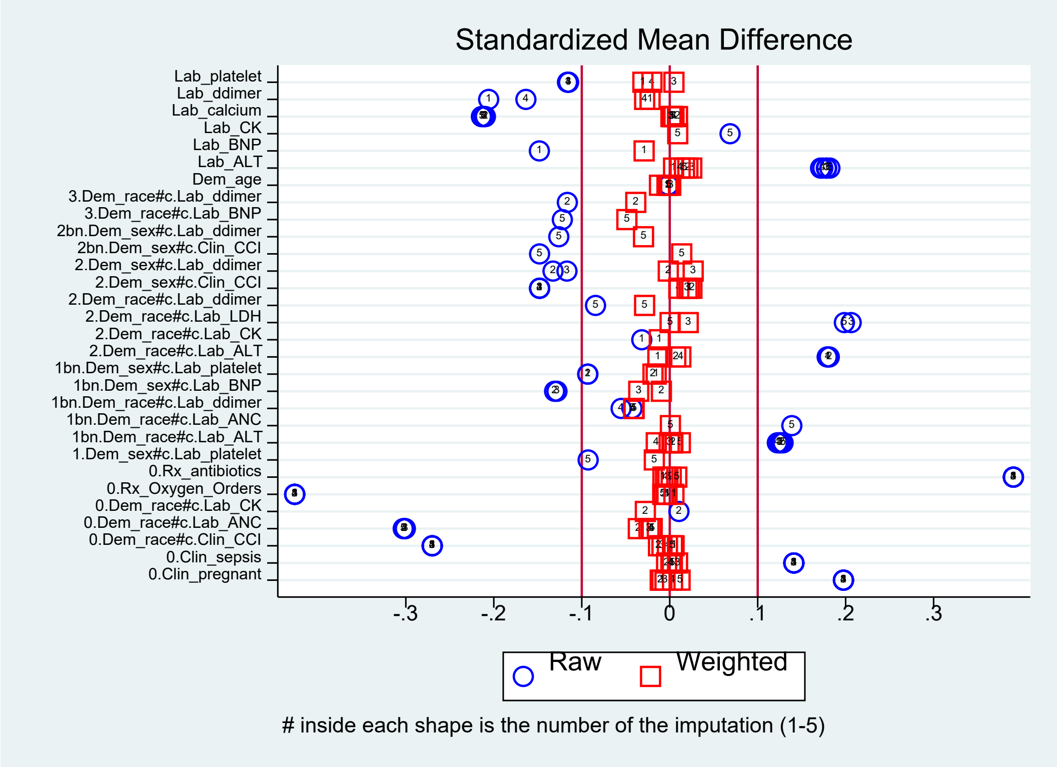

App Fig 9b

SA-Effect of FFT algorithm on outcomes: VTE

All data

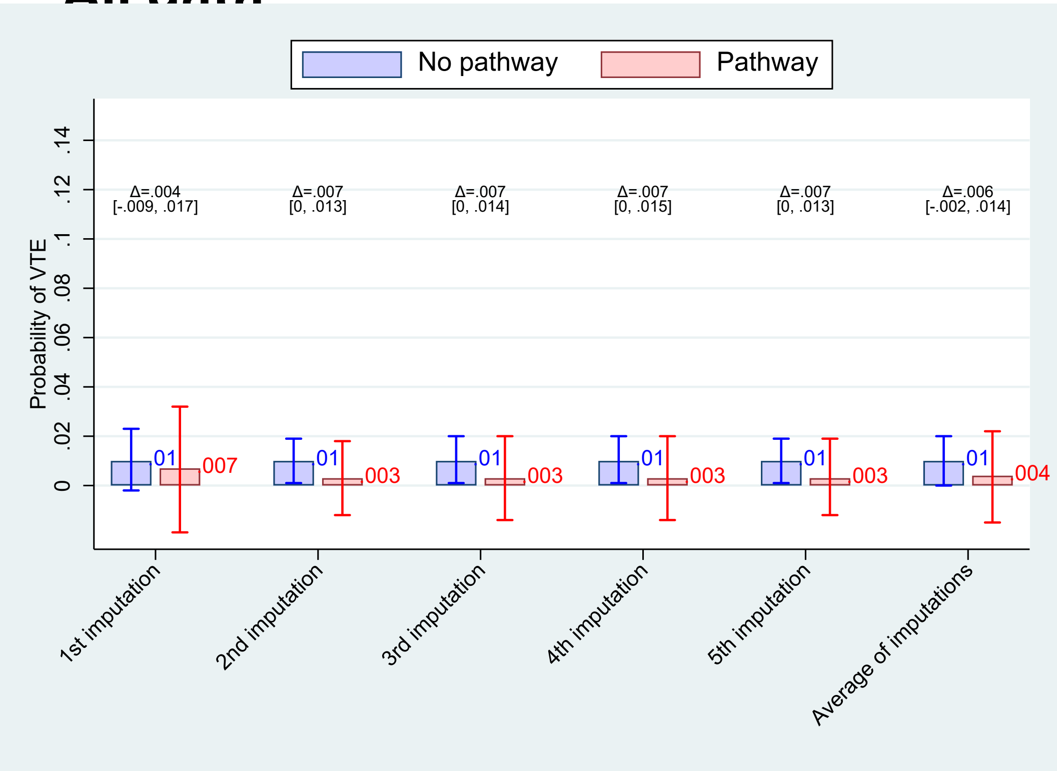

After dropping AC switch

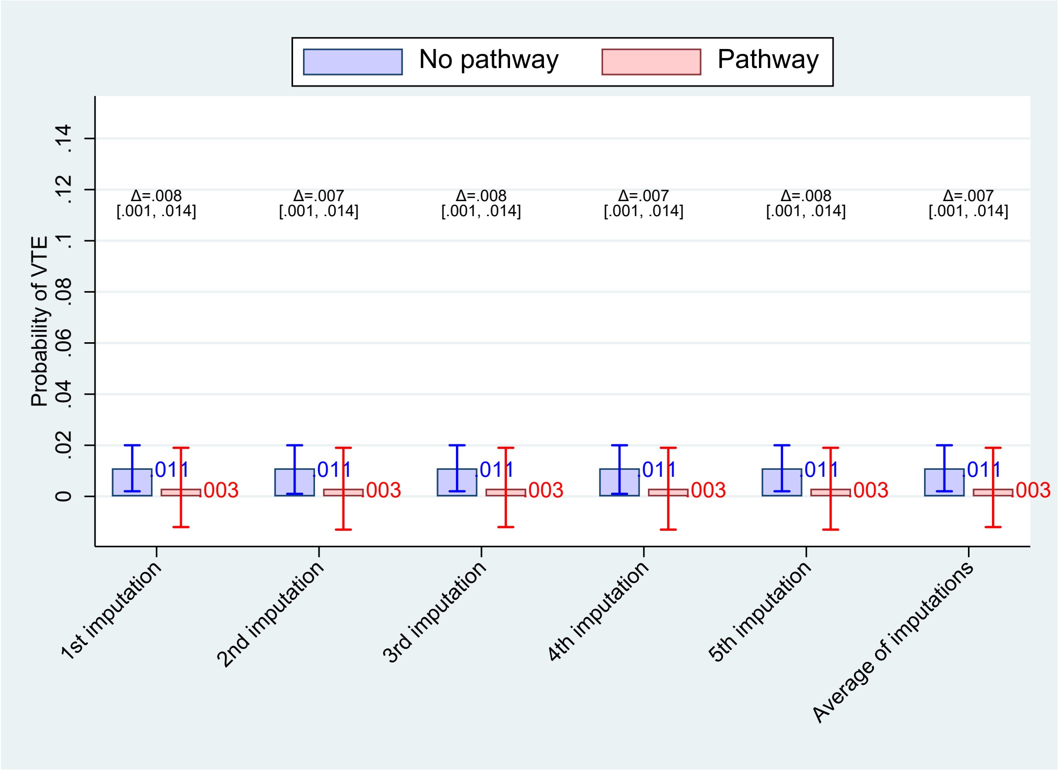

convergence could not be achieved using adaptative lasso; convergence achieved using plugin method

App Fig 10a

SA-Diagnostics for overlap assumption and for balance in the covariate distribution between treated (on FFT-algorithm) and untreated (off FFT-algorithm): VTE

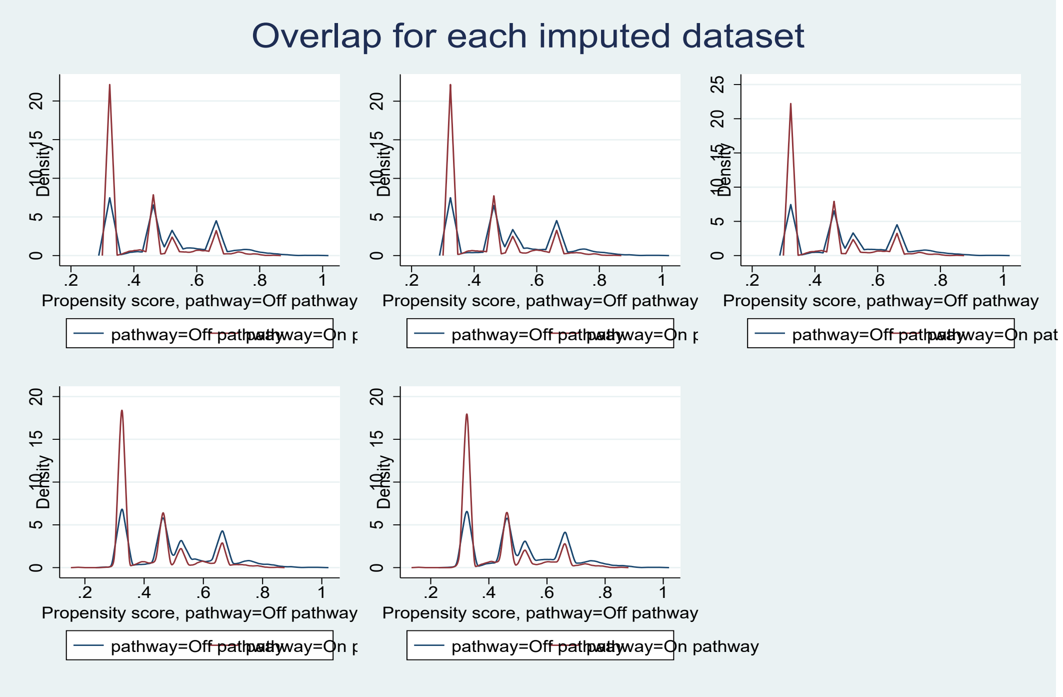

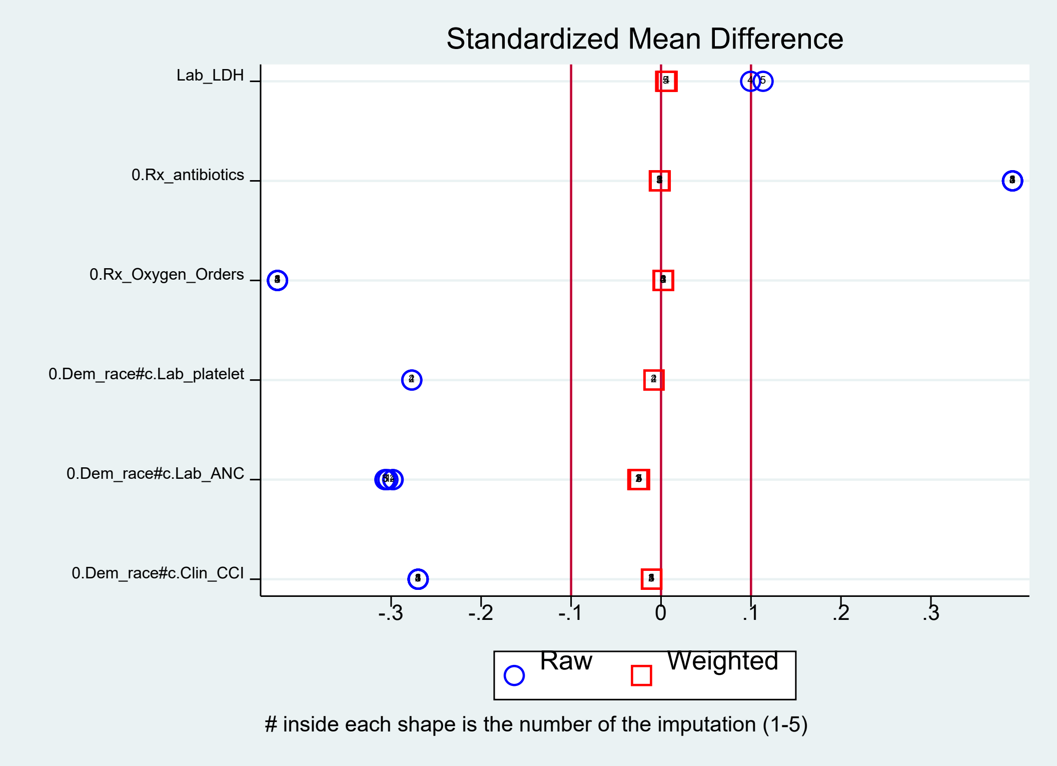

convergence could not be achieved using adaptative lasso; convergence achieved using plugin method

App Fig 10b

SA-Effect of FFT algorithm on outcomes: major bleed

All data

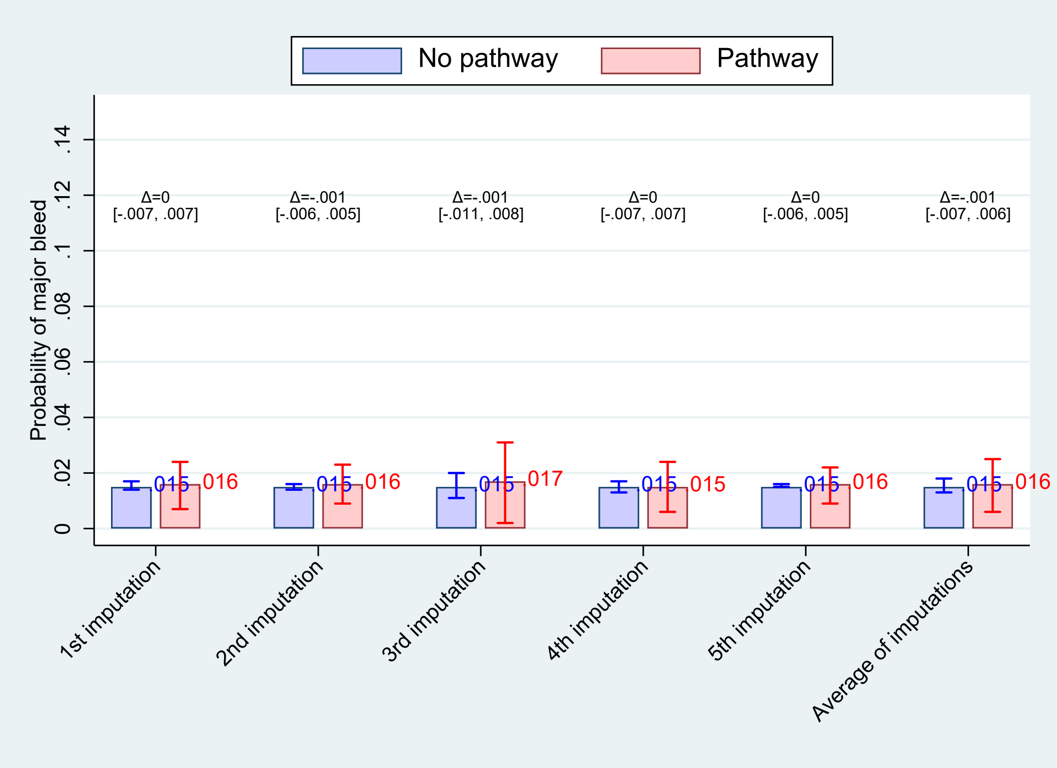

After dropping AC switch

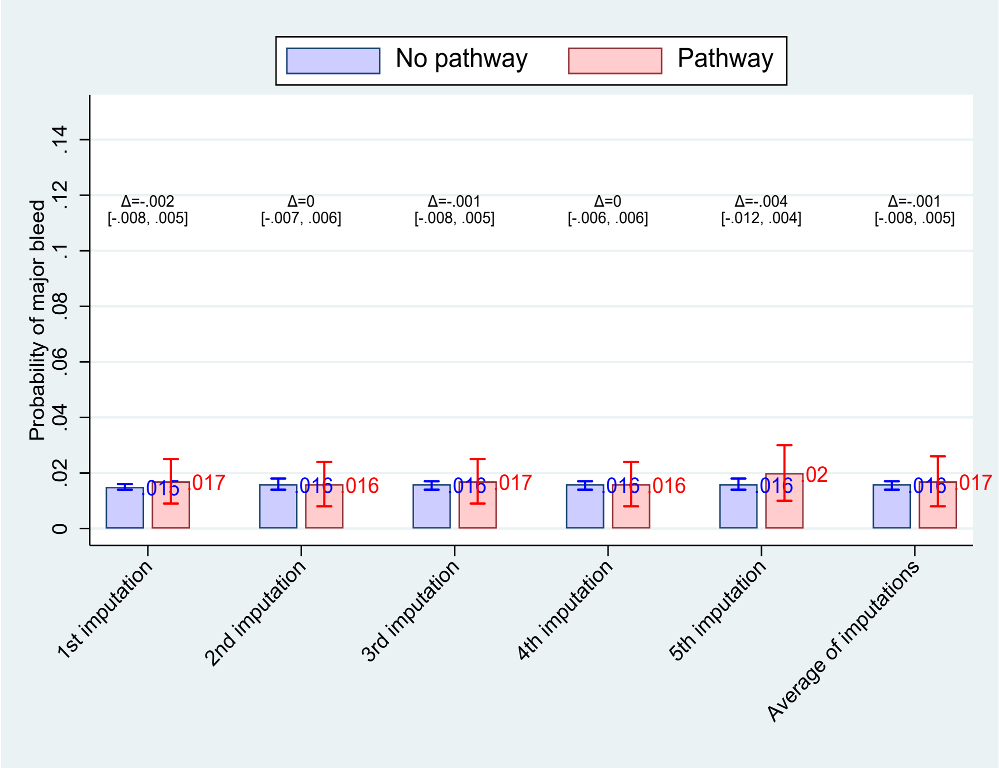

App Fig 11a

SA-Diagnostics for overlap assumption and for balance in the covariate distribution between treated (on FFT-algorithm) and untreated (off FFT-algorithm): major bleed

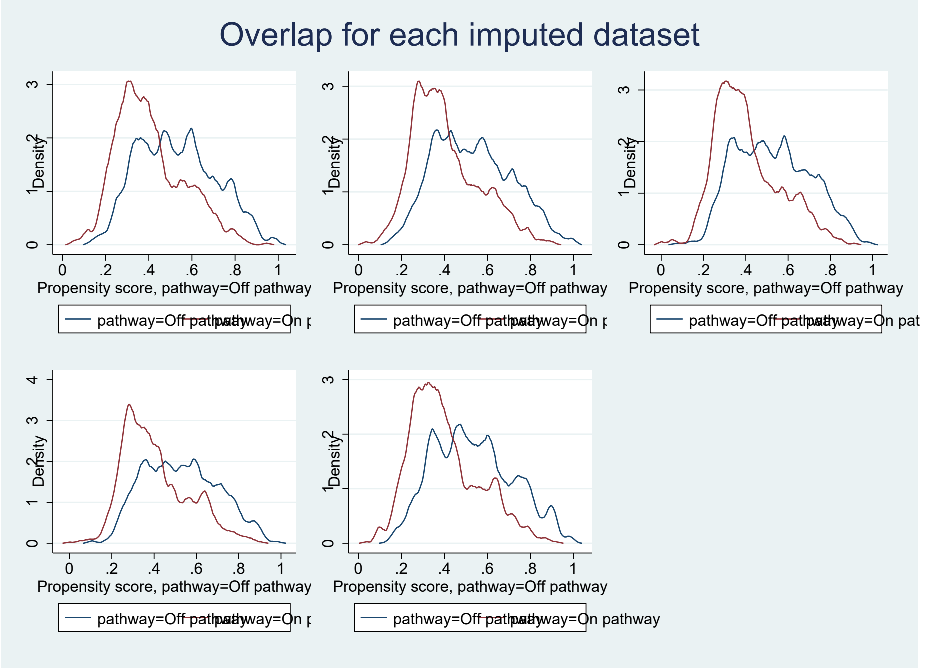

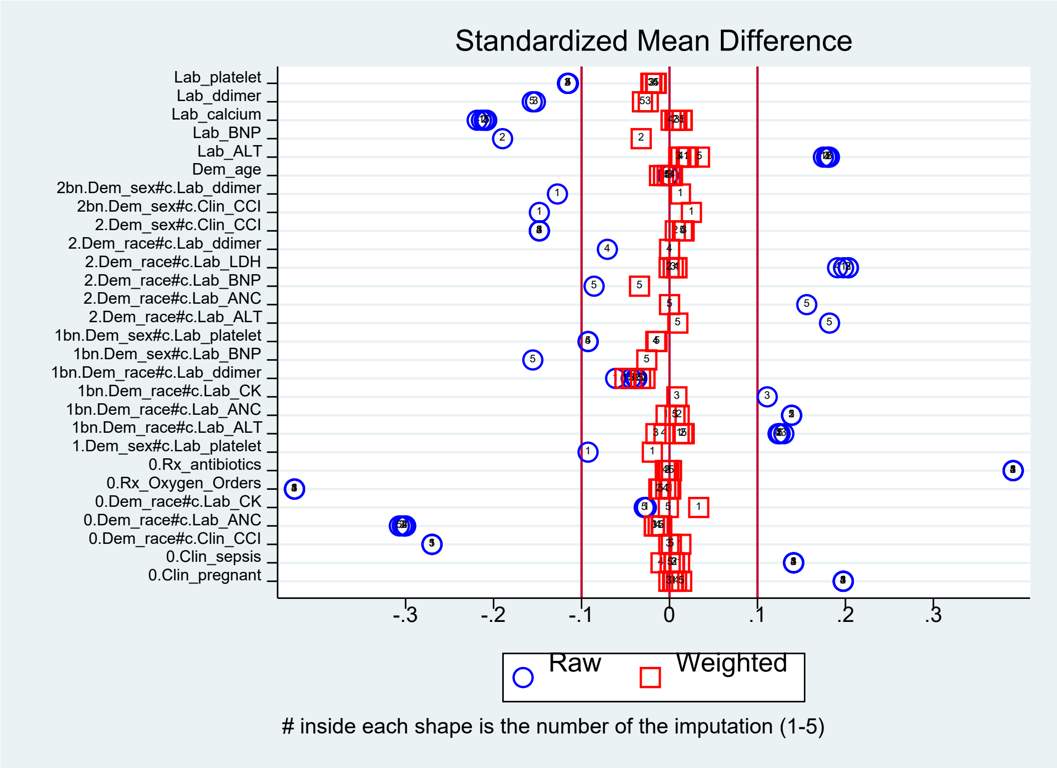

App Fig 11b

Appendix 2

App2_Tables 1,2 and 3

Lasso-propensity score regression analysis showing pathway-FFT algorithm (vs. management off pathway) on primary outcome: **death, venous thromboembolism (VTE) and major bleed.**

The analysis displays ATE [The average treatment effect= the mean of the difference between outcomes of patients treated on - off pathway-FFT algorithm] as well as all regression coefficients selected by lasso for each imputation shown; as described in the manuscript, we used Rubin's rules to combine the imputed data sets into a final pooled estimate (see Fig 3, and Figures in Appendix #1)

App Table 1: Telasso Regression analysis: effect of pathway-FFT algorithm on outcome: death#

1st imputation

Out_Death	Coefficient	Robust std. err.	z	P>|z|	[95% conf. interval]	
ATE*						
pathway (On pathway vs Off pathway)	-.0020224	.0041138	-0.49	0.623	-.0100853	.0060405
POmean						
pathway						
Off pathway	.0261705	.0081236	3.22	0.001	.0102485	.0420924

	Out_Death(0)	Out_Death(1)	pathway
Lab_ANC	1.076		
Dem_age	1.043	1.141	1.014
Clin_BMI	0.927		
Clin_CCI	1.213		
0.Clin_cultures_pos	0.412		
0.Rx_antibiotics	0.145	0.226	1.790
0.Rx_antivirals	2.227		
0bn.Dem_race#c.Lab_ANC	1.058		0.948
3.Dem_race#c.Lab_ANC		1.274	
1.Dem_race#c.Lab_ANC			1.055
0bn.Dem_race#c.Lab_LDH	1.003		
2.Dem_race#c.Lab_LDH			1.001
2.Dem_race#c.Clin_CCI	1.290		
0bn.Dem_race#c.Clin_CCI			0.946
0.Rx_SuppO2		0.219	
2bn.Dem_sex#c.Clin_CCI		1.235	0.876
Lab_ALT			1.003
Lab_calcium			0.738
Lab_ddimer			0.972
0.Clin_pregnant			2.571
0.Clin_sepsis			1.607
0.Rx_Oxygen_Orders			0.515
2bn.Dem_sex#c.Lab_ddimer			0.920
1.Dem_sex#c.Lab_platelet			0.998
_cons	0.004	0.000	2.093

2nd imputation

Out_Death	Coefficient	Robust std. err.	z	P>|z|	[95% conf. interval]	
ATE						
pathway (On pathway vs Off pathway)	-.0008931	.0053405	-0.17	0.867	-.0113602	.0095741
POmean						
pathway Off pathway	.0262585	.0080683	3.25	0.001	.010445	.042072

	Out_Death(0)	Out_Death(1)	pathway
Lab_ANC	1.078		
Dem_age	1.052	1.141	1.013
Clin_BMI	0.916		
Clin_CCI	1.242		
0.Clin_cultures_pos	0.335		
0.Rx_antibiotics	0.117	0.200	1.821
0.Rx_antivirals	2.667		
0bn.Dem_race#c.Lab_ANC	1.119		0.949
3.Dem_race#c.Lab_ANC		1.291	
1.Dem_race#c.Lab_ANC			1.044
0bn.Dem_race#c.Lab_CK	1.001		
2.Dem_race#c.Lab_CK			0.999
0bn.Dem_race#c.Lab_BNP	1.001		
2.Dem_race#c.Lab_BNP	1.001		
1bn.Dem_sex#c.Lab_ddimer	0.729		
2.Dem_sex#c.Clin_CCI	1.011	1.300	0.901
Lab_CK		0.999	
0.Rx_Oxygen_Orders		0.434	0.517
0.Rx_SuppO2		0.264	
Lab_ALT			1.002
Lab_calcium			0.745
Lab_ddimer			0.958
Lab_platelet			0.999
Lab_BNP			1.000
0.Clin_pregnant			2.720
0.Clin_sepsis			1.532
0.Rx_steroids			0.820
1.Dem_race#c.Lab_ALT			1.003
2.Dem_race#c.Lab_ALT			1.001
2.Dem_race#c.Lab_LDH			1.001
1bn.Dem_sex#c.Lab_BNP			0.999
0bn.Dem_race#c.Clin_CCI			0.941
_cons	0.004	0.000	2.301

3rd imputation

Out_Death	Coefficient	Robust std. err.	z	P>|z|	[95% conf. interval]	
ATE pathway (On pathway vs Off pathway)	-.0072832	.0039297	-1.85	0.064	-.0149853	.0004189
POmean pathway Off pathway	.0277195	.008075	3.43	0.001	.0118927	.0435463

	Out_Death(0)	Out_Death(1)	pathway
Lab_ANC	1.065		
Dem_age	1.052		1.013
Clin_CCI	1.251		
0.Clin_cultures_pos	0.284		
0.Rx_antibiotics	0.142		1.841
0bn.Dem_race#c.Lab_ANC	1.063		0.923
0bn.Dem_race#c.Lab_CK	1.002		1.001
Lab_ALT			1.001
Lab_calcium			0.741
0.Clin_pregnant			2.362
0.Clin_sepsis			1.511
0.Rx_Oxygen_Orders			0.515
0.Rx_steroids			0.833
1.Dem_race#c.Lab_ALT			1.007
2.Dem_race#c.Lab_ALT			1.003
0bn.Dem_race#c.Lab_BNP			0.999
2bn.Dem_sex#c.Lab_ddimer			0.926
1.Dem_sex#c.Lab_platelet			0.998
1.Dem_sex#c.Lab_BNP			1.000
0bn.Dem_race#c.Clin_CCI			0.949
2bn.Dem_sex#c.Clin_CCI			0.869
_cons	0.001	0.025	2.616

4th imputation

Out_Death	Coefficient	Robust std. err.	z	P>|z|	[95% conf. interval]	
ATE pathway (On pathway vs Off pathway)	.0004358	.0039588	0.11	0.912	-.0073232	.0081948
POmean pathway Off pathway	.0257229	.0086628	2.97	0.003	.0087441	.0427017

	Out_Death(0)	Out_Death(1)	pathway
Lab_ANC	1.065		
Dem_age	1.073	1.119	1.012
Clin_CCI	1.213		
0.Clin_cultures_pos	0.298		
0.Rx_antibiotics	0.107	0.222	1.796
0bn.Dem_race#c.Lab_ANC	1.150		0.958
3.Dem_race#c.Lab_ANC		1.258	
0bn.Dem_race#c.Lab_CK	1.001		
2.Dem_race#c.Lab_CK		1.001	
2.Dem_race#c.Lab_BNP	1.001		
1.Dem_race#c.Lab_BNP		1.002	
1bn.Dem_sex#c.Lab_ddimer	0.671		
1bn.Dem_sex#c.Lab_BNP		1.002	1.000
2.Dem_sex#c.Clin_CCI		1.225	0.904
Lab_ALT			1.002
Lab_calcium			0.723
Lab_ddimer			0.951
Lab_platelet			0.999
Lab_BNP			1.000
0.Clin_pregnant			2.766
0.Clin_sepsis			1.511
0.Rx_Oxygen_Orders			0.507
1.Dem_race#c.Lab_ALT			1.007
2.Dem_race#c.Lab_ALT			1.002
2.Dem_race#c.Lab_LDH			1.000
0bn.Dem_race#c.Clin_CCI			0.936
_cons	0.000	0.000	2.829

5th imputation

Out_Death	Coefficient	Robust std. err.	z	P>|z|	[95% conf. interval]	
ATE pathway (On pathway vs Off pathway)	-.0021151	.0034055	-0.62	0.535	-.0087899	.0045596
POmean pathway Off pathway	.0251181	.0085277	2.95	0.003	.0084041	.041832

	Out_Death(0)	Out_Death(1)	pathway
Lab_ANC	1.074		
Dem_age	1.072	1.102	1.013
Clin_CCI	1.267	1.290	
0.Clin_cultures_pos	0.282		
0.Rx_antibiotics	0.183		1.793
0bn.Dem_race#c.Lab_ANC	1.063		0.937
0bn.Dem_race#c.Lab_LDH	1.003		

2.Dem_race#c.Lab_BNP	1.002		
0bn.Dem_race#c.Lab_BNP			0.999
Lab_ALT			1.001
Lab_calcium			0.726
Lab_ddimer			0.954
0.Clin_pregnant			2.338
0.Clin_sepsis			1.586
0.Rx_Oxygen_Orders			0.513
1.Dem_race#c.Lab_ALT			1.007
2.Dem_race#c.Lab_ALT			1.003
1bn.Dem_sex#c.Lab_platelet			0.999
2.Dem_sex#c.Clin_CCI			0.862
_cons	0.000	0.000	2.881

* ATE-The average treatment effect= the mean of the difference between outcomes of patients treated on - off pathway-FFT algorithm; POmean- potential-outcome means in population of patients treated off pathway-FFT algorithm; Out-outcome

#- regression for each imputation shown; as described in the manuscript, we used Rubin's rules to combine the imputed data sets into a final pooled estimate (see Fig 3, and Figures in Appendix)

App Table 2: Telasso Regression analysis: effect of pathway-FFT algorithm on outcome: VTE#

1st imputation

Out_VTE	Coefficient	Robust std. err.	z	P>|z|	[95% conf. interval]	
ATE*						
pathway (On pathway vs Off pathway)	-.0074115	.0035474	-2.09	0.037	-.0143643	-.0004588
POmean						
pathway Off pathway	.0103436	.0048368	2.14	0.032	.0008637	.0198235

	Out_VTE(0)	Out_VTE(1)	pathway
Lab_calcium		0.047	0.742
0.Clin_immunocomp		0.099	
2bn.Dem_race#c.Clin_CCI		1.583	
0.Dem_race#c.Clin_CCI			0.983
Lab_BNP			1.000
0.Clin_pregnant			3.599
0.Clin_sepsis			1.510
0.Rx_Oxygen_Orders			0.529
0.Rx_antibiotics			1.831
0.Rx_steroids			0.839
1.Dem_race#c.Lab_ALT			1.006
2bn.Dem_race#c.Lab_ALT			1.004
0.Dem_race#c.Lab_ANC			0.943
2bn.Dem_sex#c.Lab_ddimer			0.919
1.Dem_sex#c.Lab_platelet			0.999
2bn.Dem_sex#c.Clin_CCI			0.928
_cons	0.010	9.1e+08	3.585

2nd imputation

Out_VTE	Coefficient	Robust std. err.	z	P>|z|	[95% conf. interval]	
ATE						

pathway (On pathway vs Off pathway)	-.0067371	.003436	-1.96	0.050	-.0134716	-2.61e-06
POmean pathway Off pathway	.0097446	.0047437	2.05	0.040	.0004472	.0190421

	Out_VTE(0)	Out_VTE(1)	pathway
1bn.Dem_sex#c.Lab_CK	1.001		
Lab_calcium		0.047	0.741
0.Clin_immunocomp		0.099	
2bn.Dem_race#c.Clin_CCI		1.582	
0.Dem_race#c.Clin_CCI			0.944
Dem_age			1.012
0.Clin_pregnant			2.330
0.Clin_sepsis			1.580
0.Rx_Oxygen_Orders			0.510
0.Rx_antibiotics			1.812
0.Rx_steroids			0.792
1.Dem_race#c.Lab_ALT			1.007
2bn.Dem_race#c.Lab_ALT			1.006
0.Dem_race#c.Lab_ANC			0.939
2bn.Dem_race#c.Lab_CK			0.999
1bn.Dem_sex#c.Lab_platelet			0.998
2.Dem_sex#c.Clin_CCI			0.861
_cons	0.007	9.1e+08	2.855

3rd imputation

Out_VTE	Coefficient	Robust std. err.	z	P>|z|	[95% conf. interval]	
ATE pathway (On pathway vs Off pathway)	.0047945	.0003704	12.94	0.000	.0040684	.0055206
POmean pathway Off pathway	.0046462	.0037258	1.25	0.212	-.0026562	.0119487

	Out_VTE(0)	Out_VTE(1)	pathway
Lab_calcium		0.000	0.737
Lab_platelet		1.273	0.999
Lab_LDH		0.907	
0.Clin_immunocomp		0.000	
0.Rx_SuppO2		0.000	
0.Rx_antivirals		8.5e+13	
3bn.Dem_race#c.Lab_ddimer		364.715	
1.Dem_race#c.Lab_ddimer			0.618
0.Dem_race#c.Clin_CCI		3.2e+04	0.939
2.Dem_race#c.Clin_CCI		3564.490	
3bn.Dem_race#c.Clin_CCI		0.000	
Lab_ALT			1.001
Dem_age			1.012
0.Clin_pregnant			2.722
0.Clin_sepsis			1.649
0.Rx_Oxygen_Orders			0.533
0.Rx_antibiotics			1.852
0.Rx_steroids			0.831
1.Dem_race#c.Lab_ALT			1.005
2.Dem_race#c.Lab_ALT			1.002
0.Dem_race#c.Lab_ANC			0.934
1.Dem_race#c.Lab_ANC			1.142
2.Dem_race#c.Lab_LDH			1.000
0.Dem_race#c.Lab_CK			1.001
0.Dem_race#c.Lab_BNP			0.999
1.Dem_race#c.Lab_BNP			0.999
1bn.Dem_sex#c.Lab_BNP			1.000
2.Dem_sex#c.Clin_CCI			0.897
_cons	0.010	2.e+150	2.237

4th imputation

Out_VTE	Coefficient	Robust std. err.	z	P>|z|	[95% conf. interval]	
ATE pathway (On pathway vs Off pathway)	-.0082549	.0040354	-2.05	0.041	-.0161642	-.0003457
POmean pathway Off pathway	.0111116	.0052283	2.13	0.034	.0008645	.0213588

	Out_VTE(0)	Out_VTE(1)	pathway
1bn.Dem_sex#c.Lab_CK	1.001		
Lab_calcium		0.047	0.710
0.Clin_immunocomp		0.099	
2bn.Dem_race#c.Clin_CCI		1.583	
Lab_ALT			1.003
Lab_ddimer			0.955
Lab_BNP			1.000
Dem_age			1.010
0.Clin_pregnant			2.894
0.Rx_Oxygen_Orders			0.511
0.Rx_antibiotics			1.812
0.Dem_race#c.Lab_ANC			0.923
2bn.Dem_race#c.Lab_LDH			1.001
2.Dem_sex#c.Clin_CCI			0.914
_cons	0.007	9.1e+08	4.119

5th imputation

Out_VTE	Coefficient	Robust std. err.	z	P>|z|	[95% conf. interval]	
ATE pathway (On pathway vs Off pathway)	-.0069147	.0033913	-2.04	0.041	-.0135615	-.0002679
POmean pathway Off pathway	.0098881	.0046635	2.12	0.034	.0007479	.0190284

	Out_VTE(0)	Out_VTE(1)	pathway
Lab_calcium		0.047	0.750
0.Clin_immunocomp		0.099	
2bn.Dem_race#c.Clin_CCI		1.583	
Lab_ALT			1.002
Dem_age			1.013
0.Clin_pregnant			2.696
0.Clin_sepsis			1.639
0.Rx_Oxygen_Orders			0.530
0.Rx_antibiotics			1.828
0.Dem_race#c.Lab_ANC			0.909
1.Dem_race#c.Lab_ANC			1.125
1.Dem_race#c.Lab_ddimer			0.734

2bn.Dem_race#c.Lab_LDH			1.001
0.Dem_race#c.Lab_CK			1.001
1bn.Dem_sex#c.Lab_platelet			0.999
1bn.Dem_sex#c.Lab_BNP			0.999
2.Dem_sex#c.Clin_CCI			0.843
_cons	0.010	9.1e+08	1.603

* ATE-The average treatment effect= the mean of the difference between outcomes of patients treated on - off pathway-FFT algorithm; POmean- potential-outcome means in population of patients treated off pathway-FFT algorithm; Out-outcome

#- regression for each imputation shown; as described in the manuscript, we used Rubin's rules to combine the imputed data sets into a final pooled estimate (see Fig 3, and Figures in Appendix)

App Table 3: Telasso Regression analysis: effect of pathway-FFT algorithm on outcome: major bleeding#

1st imputation

Out_Majorbleed	Coefficient	Robust std. err.	z	P>|z|	[95% conf. interval]	
ATE*						
pathway (On pathway vs Off pathway)	.0000939	.0034132	0.03	0.978	-.0065958	.0067836
POmean						
pathway Off pathway	.015467	.0009158	16.89	0.000	.0136721	.0172618

2nd imputation

Out_Majorbleed	Coefficient	Robust std. err.	z	P>|z|	[95% conf. interval]	
ATE						
pathway (On pathway vs Off pathway)	.0005002	.0029064	0.17	0.863	-.0051963	.0061967
POmean						
pathway Off pathway	.0152938	.000535	28.58	0.000	.0142451	.0163424

3rd imputation

Out_Majorbleed	Coefficient	Robust std. err.	z	P>|z|	[95% conf. interval]	
ATE						
pathway (On pathway vs Off pathway)	.0014724	.004918	0.30	0.765	-.0081666	.0111115
POmean						
pathway Off pathway	.0152651	.0023711	6.44	0.000	.0106177	.0199124

4th imputation

Out_Majorbleed	Coefficient	Robust std. err.	z	P>|z|	[95% conf. interval]	
ATE						
pathway (On pathway vs Off pathway)	.0001379	.003538	0.04	0.969	-.0067964	.0070722
POmean						
pathway Off pathway	.015147	.0011228	13.49	0.000	.0129463	.0173477

5th imputation

Out_Majorbleed	Coefficient	Robust std. err.	z	P>|z|	[95% conf. interval]	
ATE						
pathway (On pathway vs Off pathway)	.0004079	.0029727	0.14	0.891	-.0054185	.0062342
POmean						
pathway Off pathway	.0153956	.00028	54.98	0.000	.0148467	.0159444

* ATE-The average treatment effect= the mean of the difference between outcomes of patients treated on - off pathway-FFT algorithm; POmean- potential-outcome means in population of patients treated off pathway-FFT algorithm

#- regression for each imputation shown; as described in the manuscript, we used Rubin's rules to combine the imputed data sets into a final pooled estimate (see Fig 3, and Figures in Appendix)

Appendix 3
Information Request Analysis Details

January 19, 2022

Request Details

- Information Request: 208
- Requestor: Michael Barbee
- Title: Development of evidence-based decision support for the management of COVID19
- Analyst: Nag Tippireddy

OBJECTIVE:

- Can the development of clinical guidelines panels (CPGs) leveraging fast and frugal decision trees (FFTs) improve clinical the management and outcomes of COVID19 patients?

Requested Data:

The study requests clinical data on Rush's cohort of who were tested COVID Positive. Data are from different domains, including:

- Demographics
- Laboratory
- Medication
- Diagnosis/Condition
- Surgery/Procedures
- Other Miscellaneous data

Dataset:

The output of analysis was exported to a set of Excel spreadsheet corresponding to domains of data. The spreadsheet was posted to an OneDrive folder that the requestor shared.

The following data dictionary describes the fields in the spreadsheet.

DATA DICTIONARY

Inclusion criteria:

The dataset includes all COVID Positive patients as per the Algorithm 3.

Algorithm 3: one-sided window

Appendix 3
Information Request Analysis Details

January 19, 2022

Algorithm 3 is like Algorithm 1, except that only encounters that occur after positive results are included.

As with Algorithm 1,

- If a positive test result occurs during an encounter, the encounter is considered COVID-19 positive.
- If there is a negative result between the positive result and the admission, the encounter will be excluded.

The following columns from the dataset set namely DepartmentName, DepartmentSpecialty, AdmissionSource, and DischargeDisposition will help to filter out subsequent hospice or acute rehab encounter

Resolution:
The dataset will have one row per positive encounter for unique patient.

Data Dictionary:

Column Name	Description	Comment
MRN	Identify Unique patient	Patient MRN
Sex	Patient Sex	
Age	Patient Age	Age at the time of encounter
AgeBinned	Patient Age grouped	
Ethnicity	Patient Ethnicity	
Race	Patient Race	
Death	Death (Yes/No)	Patient live status
Pregnant	Pregnant (Yes/No)	Patient is Pregnant at the time of Encounter
COVID	Lab test indicates Positive or Negative	
Date of admission	Patient Admission Date	
Date of Discharge	Patient Discharge Date	
DepartmentName	Name of the Department	
DepartmentSpecialty	Name of the specialty	
AdmissionSource	Source patient got admitted	
DischargeDisposition	Patient discharged	
LOS_Greater_10	Length Of Stay>=10 (Yes/No)	length of stay ≥10 days then Yes

Appendix 3
Information Request Analysis Details

January 19, 2022

Column Name	Description	Comment
ICU Admission	Indicates if a patient is admitted to ICU (Yes/No)	Patient in ICU then Yes
TotalICULengthOfStay	No of ICU Days	Total number of ICU days within the encounter
Mechanical Ventilation	Mechanical Ventilation (Yes/No)	if patient has any of these recorded at positive encounter then considered as **Yes**<br>Ventilation_Flowsheets,<br>Ventilation_Orders,<br>Intubation_Flowsheets,<br>Intubation_Orders,<br>Extubation_Flowsheets,<br>Extubation_Orders,<br>LDA_name<br><br>These are variables that identify if patient was on mechanical ventilation
OSH Transfer	Transfer (Yes/No)	If Patient is transferred from other hospitals/clinics/facilities
ECMO	ECMO (Yes/No)	if patient has any of these recorded at positive encounter then considered as **Yes**<br>ECMO_Flowsheets,<br>ECMO_Orders
Oxygen_Orders	Oxygen Orders (Yes/No)	Based on any order at any point during the encounter
Height	Patient Height (cms)	Height recorded on patient encounter
Weight	Patient Weight (Kgs)	Weight recorded on patient encounter
BMI	Patient BMI	Calculated based on Height & Weight
High-flow O2	RUSH R RESPIRATORY OXYGEN FLOW RATE (l/min) (Yes/No)	Changed the column to look for Rush R Respiratory Delivery Device [3040003594] and if a patient has recorded values ‘2’ and ‘3’ considered as ‘Yes’

Appendix 3
Information Request Analysis Details

January 19, 2022

Column Name	Description	Comment
		This variable identifies if the patient was on high flow oxygen or not
Supplemental 02	RUSH R PT ROOM AIR (Yes/No)	Changed the column to look for Rush R Respiratory Oxygen Flow Rate [3040010600] and if a patient has recorded values >0 considered as 'Yes'<br><br>Identifies patients who are on supplemental oxygen, not high flow
O2_saturation_95	PULSE OXIMETRY (%) >=95% (Yes/No)	If patient encounter has pulse oximeter >=95 recorded then considered as Yes
Creatinine	Creatinine (continuous) (mg/dl)	Maximum value recorded at the time of encounter
Creatinine clearance	Creatinine clearance (continuous) (mg/dl)	Maximum value recorded at the time of encounter
Admission_Creatinin	Creatinine (continuous) (mg/dl) at the time of admission	First Creatinine level at the time of admission
Discharge_Creatinin	Creatinine (continuous) (mg/dl) at the time of discharge	Most recent Creatinine level at the time of discharge
Cultures_Positive	Category (Yes/No)	If a patient is tested **positive microbiology/blood culture data** for CBLOOD considered as 'Yes'
Ddimer	ddimer at the time of admission	First ddimer value at the time of admission
Admission (AST, AST…..)	Admission values at the time of encounter	Admission values for AST and ALT
Peak (AST, AST…..)	Peak values at the time of encounter	Peak values for AST and ALT during the encounter
Sepsis	Category (Yes/No)	A40.xx/A41.xx family ICD10 Codes with diagnosis type Encounter and Hospital Problem

Appendix 3
Information Request Analysis Details

January 19, 2022

Column Name	Description	Comment
		See Tables below for Sepsis ICD 10 codes
SepStart	Diagnosis registered date in EPIC/Patient Chart	Date when sepsis diagnosis was registered into chart<br><br>See Tables below for Sepsis ICD 10 codes used
Immunocompromised	Category (Yes/No)	D84.9 and all children, D84.821, D84.89, Z79.52, Z94.84 ICD10 Codes with diagnosis type Encounter and Hospital Problem<br><br>See table below for ICD 10 codes for Immunocompromised
ImmunoStart	Diagnosis registered date in EPIC/Patient Chart	Date when patient was registered as immunocompromised
VTE	Category (Yes/No)	I82.xx, I26.XX and I80.XX family ICD10 Codes with diagnosis type Encounter and Hospital Problem<br><br>See tables below for ICD 10 codes for VTE
VTEStart	Diagnosis registered date in EPIC/Patient Chart	Date when patient was registered with VTE diagnosis
Bleeding	Category (Yes/No)	ICD-10 codes for ‘bleeding’, ‘blood’ with diagnosis type Encounter and Hospital Problem’<br><br>See table below for ICD 10 codes for Bleeding
BleedingStart	Diagnosis registered date in EPIC/Patient Chart	Date when patient was registered with Bleeding
Heparin_induced_thrombocytopenia	Category (Yes/No)	D75.82 ICD10 Code with diagnosis type Encounter and Hospital Problem<br><br>Based on ICD 10 codes for HIT
HITStart	Diagnosis registered date in EPIC/Patient Chart	Date when patient was registered with ICD 10 code for bleeding

Appendix 3
Information Request Analysis Details

January 19, 2022

Column Name	Description	Comment
ESRD	Category (Yes/No)	`N18.6 ICD10 Code` with diagnosis type Encounter and Hospital Problem
ESRDStart	Diagnosis registered date in EPIC/Patient Chart	Date when patient was registered with ICD 10 code for ESRD
Non Major bleeding	Category (Yes/No)	`ICD 10 codes` with diagnosis type Encounter and Hospital Problem<br><br>See table below for ICD-10 codes related to non-major bleeding
NonMajorBleedingStart	Diagnosis registered date in EPIC/Patient Chart	Date when patient was registered with ICD 10 code for non-major bleeding
Major_bleeding	Category (Yes/No)	`ICD 10 codes` with diagnosis type Encounter and Hospital Problem<br><br>See table below for ICD-10 codes related to major bleeding
MajorBleedingStart	Diagnosis registered date in EPIC/Patient Chart	Date when patient was registered with ICD 10 code for Major bleeding
Remdesivir	Category (Yes/No)- Therapy	If Patient is under remdesivir medication therapy at the time of positive encounter considered as Yes<br>Included all formulations of remdesivir
Totaltheraphydays_Remdesivir	Remdesivir Duration of Therapy	Duration of therapy = the total number of remdesivir doses administered during the course of therapy
Dexamethasone	Category (Yes/No)- Therapy	If Patient is under Dexamethasone therapy medication at the time of positive encounter considered as Yes<br><br>Included all formulations of Dexamethasone (PO and IV)

Appendix 3
Information Request Analysis Details

January 19, 2022

Column Name	Description	Comment
Totaltheraphydays_Dexamethasone	Dexamethasone Duration of Therapy	Duration of therapy = the total number of Dexamethasone doses administered during the course of therapy
Dexamethasone_Remdesivir	Category (Yes/No)- Therapy	If Patient is under Remdesivir + Dexamethasone therapy at the time of positive encounter considered as Yes
Totaltheraphydays_Combination	Total Duration of Therapy (Dexamethasone+Remdesivir)	Changed to the count to minimum no of doses among Remdesivir and Dexamethasone
Heparins	Category (Yes/No)- Treatment	Drugs with PharmaceuticalSubclass-Heparin and AdministrationAction (Given, New Bag) considered as Yes and action
Dose_Heparins	Heparins Duration of Therapy	Duration of therapy = the total number of Heparins doses administered during the course of therapy
Heparin_last_dose	Dose of Heparin	Last dose of Heparin taken by the patient during the encounter
DOAC	Category (Yes/No)- Treatment	Patient has any of the listed drugs at the time of encounter and AdministrationAction (Given, New Bag) considered as Yes<br>Apixaban<br>Betrixaban<br>Dabigatran<br>Edoxaban0<br>Rivaroxaban
Dose_Apixaban	Apixaban Duration of Therapy	Duration of therapy = the total number of Apixaban doses administered during the course of therapy
Apixaban_last_dose	Dose of Apixaban	Last dose of Apixaban taken by the patient during the encounter

Appendix 3
Information Request Analysis Details

January 19, 2022

Column Name	Description	Comment
Dose_Dabigatran	Dabigatran Duration of Therapy	Duration of therapy = the total number of Dabigatran doses administered during the course of therapy
Dabigatran_last_dose	Dose of Dabigatran	Last dose of Dabigatran taken by the patient during the encounter
Dose_Betrixaban	Betrixaban Duration of Therapy	Duration of therapy = the total number of Betrixaban doses administered during the course of therapy
Betrixaban_last_dose	Dose of Betrixaban	Last dose of Betrixaban taken by the patient during the encounter
Dose_Edoxaban	Edoxaban Duration of Therapy	Duration of therapy = the total number of Edoxaban doses administered during the course of therapy
Edoxaban_last_dose	Dose of Edoxaban	Last dose of Edoxaban taken by the patient during the encounter
Dose_Rivaroxaban	Rivaroxaban Duration of Therapy	Duration of therapy = the total number of Rivaroxaban doses administered during the course of therapy
Rivaroxaban_last_dose	Dose of Rivaroxaban	Last dose of Rivaroxaban taken by the patient during the encounter
LMWH	Category (Yes/No)-Treatment	Drugs with PharmaceuticalSubclass- Low Molecular Weight Heparins and AdministrationAction (Given, New Bag) considered as Yes
Dose_LMWH	LMWH Duration of Therapy	Duration of therapy = the total number of LMWH doses administered during the course of therapy
LMWH_last_dose	Dose of LMWH	Last dose of LMWH taken by the patient during the encounter
Antibiotics	Category (Yes/No)-Treatment	Drugs with TherapeuticClass - ANTIBIOTICS and

Appendix 3
Information Request Analysis Details

January 19, 2022

Column Name	Description	Comment
		AdministrationAction (Given, New Bag) considered as Yes
Antivirals	Category (Yes/No)- Treatment	Drugs with TherapeuticClass - ANTIVIRALS and AdministrationAction (Given, New Bag) considered as Yes Included remdesivir in Antiviral drugs
Steroids	Category (Yes/No)- Treatment	Oral prednisone and dexamethasone(IV or Oral) considered and AdministrationAction (Given, New Bag) as Yes
ID_CONSULT	Category (Yes/No)	If a patient has INFECTIOUS DISEASE consult after date of admission then considered as Yes
MATERNAL_CONSULT	Category (Yes/No)	If a patient MATERNAL CONSULT after date of admission then considered as Yes
Charlson Comorbidity Index	Charlson score	Calculated based on https://www.mdcalc.com/charlson-comorbidity-index-cci
Lovenox	Category (Yes/No)- Treatment	Drugs with simple generic name = Enoxaparin and Therapeutic Class= ANTICOAGULANTS considered as Yes
Lovenox Therapy	Therapy category	The lovenox indication of use i.e., treatment or prophylaxis

New Labs included (admission value and peak value):

1. AST
2. ALT
3. Direct bilirubin
4. LDH
5. ANC
6. Albumin
7. PTT
8. BUN
9. Calcium

Appendix 3
Information Request Analysis Details

January 19, 2022

10. CK
11. Ferritin
12. Platelet
13. BNP

Major bleeding ICD 10 Code:

ICD10	Term
K66.1	Hemoperitoneum
I31.0	Chronic adhesive pericarditis
I60.9	Nontraumatic subarachnoid hemorrhage, unspecified (CMS-HCC)
I31.4	Cardiac tamponade
I31.9	Disease of pericardium, unspecified
I61.9	Nontraumatic intracerebral hemorrhage, unspecified (CMS-HCC)
E27.40	Unspecified adrenocortical insufficiency (CMS-HCC)
H44.819	Hemophthalmos, unspecified eye
I62.1	Nontraumatic extradural hemorrhage (CMS-HCC)
I31.8	Other specified diseases of pericardium
I31.1	Chronic constrictive pericarditis
I62.00	Nontraumatic subdural hemorrhage, unspecified (CMS-HCC)
I31.2	Hemopericardium, not elsewhere classified
E27.1	Primary adrenocortical insufficiency (CMS-HCC)
H35.60	Retinal hemorrhage, unspecified eye
I62.9	Nontraumatic intracranial hemorrhage, unspecified (CMS-HCC)
E27.2	Addisonian crisis (CMS-HCC)

Non- Major bleeding ICD 10 Code:

ICD10	Term
R04.1	Hemorrhage from throat
R58	Hemorrhage, not elsewhere classified
K76.1	Chronic passive congestion of liver
K26.0	Acute duodenal ulcer with hemorrhage
K86.1	Other chronic pancreatitis
K25.6	Chronic or unspecified gastric ulcer with both hemorrhage and perforation
K29.91	Gastroduodenitis, unspecified, with bleeding
K94.09	Other complications of colostomy

Appendix 3
Information Request Analysis Details

January 19, 2022

I85.01	Esophageal varices with bleeding (CMS-HCC)
R04.2	Hemoptysis
M25.00	Hemarthrosis, unspecified joint
K57.31	Diverticulosis of large intestine without perforation or abscess with bleeding
K29.51	Unspecified chronic gastritis with bleeding
M25.076	Hemarthrosis, unspecified foot
S36.029A	Unspecified contusion of spleen, initial encounter
K31.89	Other diseases of stomach and duodenum
K05.5	Other periodontal diseases
K29.60	Other gastritis without bleeding
K64.5	Perianal venous thrombosis
I85.11	Secondary esophageal varices with bleeding (CMS-HCC)
K28.2	Acute gastrojejunal ulcer with both hemorrhage and perforation (CMS-HCC)
M25.073	Hemarthrosis, unspecified ankle
S36.112A	Contusion of liver, initial encounter
K64.9	Unspecified hemorrhoids
K29.81	Duodenitis with bleeding
K13.70	Unspecified lesions of oral mucosa
K26.4	Chronic or unspecified duodenal ulcer with hemorrhage
K62.5	Hemorrhage of anus and rectum
K27.0	Acute peptic ulcer, site unspecified, with hemorrhage
R47.01	Aphasia
K26.6	Chronic or unspecified duodenal ulcer with both hemorrhage and perforation
K29.41	Chronic atrophic gastritis with bleeding
N00.9	Acute nephritic syndrome with unspecified morphologic changes
K22.6	Gastro-esophageal laceration-hemorrhage syndrome
K64.4	Residual hemorrhoidal skin tags
K25.2	Acute gastric ulcer with both hemorrhage and perforation (CMS-HCC)
K57.11	Diverticulosis of small intestine without perforation or abscess with bleeding
R31.0	Gross hematuria
K66.1	Hemoperitoneum
K57.33	Diverticulitis of large intestine without perforation or abscess with bleeding
K29.61	Other gastritis with bleeding
K06.1	Gingival enlargement
M26.79	Other specified alveolar anomalies

K06.2	Gingival and edentulous alveolar ridge lesions associated with trauma
K76.89	Other specified diseases of liver
K25.4	Chronic or unspecified gastric ulcer with hemorrhage
K28.4	Chronic or unspecified gastrojejunal ulcer with hemorrhage
K29.71	Gastritis, unspecified, with bleeding
M79.81	Nontraumatic hematoma of soft tissue
I88.1	Chronic lymphadenitis, except mesenteric
K64.8	Other hemorrhoids
R31.1	Benign essential microscopic hematuria
R04.89	Hemorrhage from other sites in respiratory passages
K25.0	Acute gastric ulcer with hemorrhage
K29.90	Gastroduodenitis, unspecified, without bleeding
K22.11	Ulcer of esophagus with bleeding
K29.50	Unspecified chronic gastritis without bleeding
K29.00	Acute gastritis without bleeding
K57.13	Diverticulitis of small intestine without perforation or abscess with bleeding
K29.80	Duodenitis without bleeding
K27.4	Chronic or unspecified peptic ulcer, site unspecified, with hemorrhage
R04.9	Hemorrhage from respiratory passages, unspecified
N32.89	Other specified disorders of bladder
I86.8	Varicose veins of other specified sites
K28.0	Acute gastrojejunal ulcer with hemorrhage
K13.79	Other lesions of oral mucosa
I85.10	Secondary esophageal varices without bleeding (CMS-HCC)
K29.40	Chronic atrophic gastritis without bleeding
K29.70	Gastritis, unspecified, without bleeding
K92.0	Hematemesis
K31.82	Dieulafoy lesion (hemorrhagic) of stomach and duodenum
K29.21	Alcoholic gastritis with bleeding
K29.20	Alcoholic gastritis without bleeding
K27.6	Chronic or unspecified peptic ulcer, site unspecified, with both hemorrhage and perforation (CMS-HCC)
N89.8	Other specified noninflammatory disorders of vagina
R31.9	Hematuria, unspecified
K29.01	Acute gastritis with bleeding
M25.069	Hemarthrosis, unspecified knee

Sepsis ICD10 Codes:

ICD10	Term

A41.02	Sepsis due to methicillin resistant Staphylococcus aureus (CMS-HCC)
A41.89	Other specified sepsis (CMS-HCC)
A41.9	Sepsis, unspecified organism (CMS-HCC)

Immunocompromised ICD10 Codes:

ICD10	Term
C92.10	Chronic myeloid leukemia, BCR/ABL-positive, not having achieved remission (CMS-HCC)
D84.821	Immunodeficiency due to drugs
C88.0	Waldenstrom macroglobulinemia (CMS-HCC)
C83.30	Diffuse large B-cell lymphoma, unspecified site (CMS-HCC)
C91.10	Chronic lymphocytic leukemia of B-cell type not having achieved remission (CMS-HCC)
C92.02	Acute myeloblastic leukemia, in relapse (CMS-HCC)
Z94.83	Pancreas transplant status (CMS-HCC)
C90.00	Multiple myeloma not having achieved remission (CMS-HCC)
C83.10	Mantle cell lymphoma, unspecified site (CMS-HCC)
C85.80	Other specified types of non-hodgkin lymphoma, unspecified site (CMS-HCC)
C83.38	Diffuse large b-cell lymphoma, lymph nodes of multiple sites (CMS-HCC)
Z94.81	Bone marrow transplant status (CMS-HCC)
Z94.84	Stem cells transplant status (CMS-HCC)
D84.9	Immunodeficiency, unspecified (CMS-HCC)
Z94.4	Liver transplant status (CMS-HCC)
C91.00	Acute lymphoblastic leukemia not having achieved remission (CMS-HCC)
C92.01	Acute myeloblastic leukemia, in remission (CMS-HCC)
Z94.0	Kidney transplant status

VTE ICD10 Codes:

ICD10	Term
I82.421	Acute embolism and thrombosis of right iliac vein (CMS-HCC)
I80.203	Phlebitis and thrombophlebitis of unspecified deep vessels of lower extremities, bilateral (CMS-HCC)
I26.92	Saddle embolus of pulmonary artery without acute cor pulmonale (CMS-HCC)
I80.201	Phlebitis and thrombophlebitis of unspecified deep vessels of right lower extremity (CMS-HCC)

I80.209	Phlebitis and thrombophlebitis of unspecified deep vessels of unspecified lower extremity (CMS-HCC)
I82.413	Acute embolism and thrombosis of femoral vein, bilateral (CMS-HCC)
I80.232	Phlebitis and thrombophlebitis of left tibial vein (CMS-HCC)
I82.401	Acute embolism and thrombosis of unspecified deep veins of right lower extremity (CMS-HCC)
I82.411	Acute embolism and thrombosis of right femoral vein (CMS-HCC)
I82.419	Acute embolism and thrombosis of unspecified femoral vein (CMS-HCC)
I82.403	Acute embolism and thrombosis of unspecified deep veins of lower extremity, bilateral (CMS-HCC)
I82.429	Acute embolism and thrombosis of unspecified iliac vein (CMS-HCC)
I26.09	Other pulmonary embolism with acute cor pulmonale (CMS-HCC)
I82.412	Acute embolism and thrombosis of left femoral vein (CMS-HCC)
I82.4Y3	Acute embolism and thrombosis of unspecified deep veins of proximal lower extremity, bilateral (CMS-HCC)
I80.10	Phlebitis and thrombophlebitis of unspecified femoral vein (CMS-HCC)
I80.13	Phlebitis and thrombophlebitis of femoral vein, bilateral (CMS-HCC)
I82.402	Acute embolism and thrombosis of unspecified deep veins of left lower extremity (CMS-HCC)
I82.422	Acute embolism and thrombosis of left iliac vein (CMS-HCC)
I82.423	Acute embolism and thrombosis of iliac vein, bilateral (CMS-HCC)
I82.431	Acute embolism and thrombosis of right popliteal vein (CMS-HCC)
I82.432	Acute embolism and thrombosis of left popliteal vein (CMS-HCC)
I82.433	Acute embolism and thrombosis of popliteal vein, bilateral (CMS-HCC)

I82.4Y2	Acute embolism and thrombosis of unspecified deep veins of left proximal lower extremity (CMS-HCC)
I82.4Y9	Acute embolism and thrombosis of unspecified deep veins of unspecified proximal lower extremity (CMS-HCC)
I26.99	Other pulmonary embolism without acute cor pulmonale (CMS-HCC)
I80.202	Phlebitis and thrombophlebitis of unspecified deep vessels of left lower extremity (CMS-HCC)
I82.409	Acute embolism and thrombosis of unspecified deep veins of unspecified lower extremity (CMS-HCC)
I82.439	Acute embolism and thrombosis of unspecified popliteal vein (CMS-HCC)
I82.4Y1	Acute embolism and thrombosis of unspecified deep veins of right proximal lower extremity (CMS-HCC)
I26.02	Saddle embolus of pulmonary artery with acute cor pulmonale (CMS-HCC)
I80.11	Phlebitis and thrombophlebitis of right femoral vein (CMS-HCC)
I80.12	Phlebitis and thrombophlebitis of left femoral vein (CMS-HCC)
I80.211	Phlebitis and thrombophlebitis of right iliac vein (CMS-HCC)
I80.292	Phlebitis and thrombophlebitis of other deep vessels of left lower extremity (CMS-HCC)

Bleeding ICD10 Codes:

ICD10	Term
O99.113	Other diseases of the blood and blood-forming organs and certain disorders involving the immune mechanism complicating pregnancy, third trimester
R03.0	Elevated blood-pressure reading, without diagnosis of hypertension
K29.71	Gastritis, unspecified, with bleeding
K57.90	Diverticulosis of intestine, part unspecified, without perforation or abscess without bleeding
R78.89	Finding of other specified substances, not normally found in blood
R79.9	Abnormal finding of blood chemistry, unspecified
D72.828	Other elevated white blood cell count
K29.20	Alcoholic gastritis without bleeding

Appendix 3
Information Request Analysis Details
January 19, 2022

R79.89	Other specified abnormal findings of blood chemistry
D72.829	Elevated white blood cell count, unspecified
K29.50	Unspecified chronic gastritis without bleeding
K50.911	Crohn's disease, unspecified, with rectal bleeding (CMS-HCC)
K57.40	Diverticulitis of both small and large intestine with perforation and abscess without bleeding
Z67.91	Unspecified blood type, rh negative
D72.9	Disorder of white blood cells, unspecified
K20.90	Esophagitis, unspecified without bleeding
K29.30	Chronic superficial gastritis without bleeding
K57.92	Diverticulitis of intestine, part unspecified, without perforation or abscess without bleeding
K20.80	Other esophagitis without bleeding
N93.9	Abnormal uterine and vaginal bleeding, unspecified
Z86.2	Personal history of diseases of the blood and blood-forming organs and certain disorders involving the immune mechanism
D72.819	Decreased white blood cell count, unspecified
I85.11	Secondary esophageal varices with bleeding (CMS-HCC)
K57.20	Diverticulitis of large intestine with perforation and abscess without bleeding
D50.0	Iron deficiency anemia secondary to blood loss (chronic)
R79.81	Abnormal blood-gas level

Heparin induced thrombocytopenia ICD10 Codes:

ICD10	Term
D75.82	Heparin induced thrombocytopenia (HIT) (CMS-HCC)

Cultures considered as Positive:

Culture
Cryptococcus neoformans
Gram stain: Gram positive cocci
Gram stain: Gram variable coccobacilli seen in Anaerobic
Pseudomonas aeruginosa
Streptococcus anginosus group
Gram positive cocci in clusters
Gram stain:
Staphylococcus aureus
Streptococcus salivarius
Streptococcus group G

Appendix 3
Information Request Analysis Details

January 19, 2022

Gram positive rods seen in Aerobic bottle
Gram stain: Gram positive cocci in pairs, chains and clusters
Gram stain: Gram positive rods seen in Aerobic bottle
Non-hemolytic Streptococci species
Candida glabrata
Candida tropicalis
Dermabacter species
yeast in Aerobic bottle
Corynebacterium species NOT C. jeikeium
Gram stain: budding yeast seen in Aerobic bottle
Growth of Gram positive cocci in pairs and chains
Klebsiella pneumoniae
Prevotella oralis
Citrobacter koseri
Gram stain: Gram positive cocci in clusters
Gram stain: Gram positive rods seen in Anaerobic bottle
Bacillus species NOT anthracis
Escherichia coli
Growth of Gram positive rods
Haemophilus influenzae
Staphylococcus epidermidis
Enterobacter aerogenes
Enterobacter cloacae
Enterococcus faecalis
Gram stain: Gram positive cocci seen in Anaerobic bottle
Growth of Budding yeast
Klebsiella pneumoniae ssp pneumoniae
Pasteurella multocida
Streptococcus pneumoniae
Anaerobe
Diphtheroids
Gram stain: Gram positive cocci in pairs and chains
Growth of Gram positive cocci in clusters
Methicillin Resistant Staphylococcus aureus
Micrococcus species
Staphylococcus hominis
Candida albicans
Cutibacterium acnes
Gram stain: Gram positive cocci in pairs and clusters
Gram stain: Gram positive cocci in pairs and clusters seen in

Appendix 3
Information Request Analysis Details

January 19, 2022

Generic Names of Drugs:

Heparins	DOAC	LMWH	Steroids
heparin sodium, porcine/D5W	dabigatran etexilate mesylate	enoxaparin sodium	methylprednisolone acetate
heparin sodium, porcine/NS/PF	apixaban		prednisolone sodium phosphate
heparin sod, pork in 0.45% NaCl	rivaroxaban		ciprofloxacin HCl/dexameth
heparin sodium, porcine/PF			methylprednisolone
heparin sodium, porcine			prednisolone
			dexamethasone sodium phosp/PF
			methylprednisolone sod succ/PF
			dexamethasone
			dexamethasone sodium phosphate

Generic Names of Antiviral Drugs:

Antivirals
dolutegravir sodium
remdesivir
nevirapine
sofosbuvir/velpatasvir
acyclovir sodium
valganciclovir HCl
acyclovir
raltegravir potassium
abacavir/dolutegravir/lamivudi
emtricitabine/tenofov alafenam
lamivudine
abacavir sulfate

Generic Names of Antibiotic Drugs:

Appendix 3
Information Request Analysis Details

January 19, 2022

elviteg/cob/emtri/tenof alafen
bictegrav/emtricit/tenofov ala
tenofovir disoproxil fumarate
entecavir
valacyclovir HCl
lopinavir/ritonavir
emtricitabine
emtricitabine/tenofovir (TDF)
oseltamivir phosphate
tenofovir alafenamide

**Antibiotics**
cefazolin sodium/D5W
erythromycin base
ethambutol HCl
penicillin V potassium
silver sulfadiazine
cefadroxil
cephalexin
linezolid in 0.9% sodium chlor
polymyxin B sulf/trimethoprim
rifaximin
cefpodoxime proxetil
clarithromycin
clindamycin HCl
levofloxacin in dextrose 5 %
gentamicin in NaCl, iso-osm
metronidazole/sodium chloride
moxifloxacin HCl
ofloxacin
penicillin G benzathine
ampicillin sodium
ciprofloxacin HCl
clindamycin in 0.9 % sod chlor
dapsone
metronidazole
nitrofurantoin monohyd/m-cryst
tetracycline HCl
tobramycin
vancomycin HCl
doxycycline hyclate
gentamicin sulfate
isoniazid
levofloxacin
cefdinir
cefepime HCl
ciprofloxacin HCl/dexameth
doxycycline monohydrate
mupirocin
cefazolin sodium
cefazolin sodium/dextrose,iso

Appendix 3
Information Request Analysis Details

January 19, 2022

cefoxitin sodium
vancomycin HCl in 5 % dextrose
amikacin sulfate
clindamycin phosphate
meropenem
nitrofurantoin macrocrystal
piperacillin-tazo-dextrose,iso
pyrazinamide
ampicillin sodium/sulbactam Na
ceftriaxone sodium
clindamycin phosphate/D5W
linezolid
rifampin
sulfamethoxazole/trimethoprim
amoxicillin
azithromycin
bacitracin zinc
cefazolin sodium in 0.9 % NaCl
ceftazidime
ertapenem sodium
neomycin/bacitracin/polymyxinB
amoxicillin/potassium clav
bacitracin
cefuroxime axetil
ciprofloxacin in 5 % dextrose
mupirocin calcium
neomycin/polymyxin B/dexametha
tobramycin in 0.225% sod chlor
vancomycin/0.9 % sod chloride
